# Pioneering electrochemical detection unveils erdafitinib: a breakthrough in anticancer agent determination

**DOI:** 10.1007/s00604-024-06318-z

**Published:** 2024-03-27

**Authors:** Merve Hatun Yildir, Asena Ayse Genc, Nevin Erk, Wiem Bouali, Nesrin Bugday, Sedat Yasar, Ozgur Duygulu

**Affiliations:** 1 Faculty of Pharmacy, Department of Analytical Chemistry, Ankara University, 06560 Ankara, Turkey; 2 Graduate School of Health Sciences, Ankara University, 06110 Ankara, Turkey; 3 Department of Chemistry, İnonu University, 44280 Malatya, Turkey; 4 TÜBİTAK Marmara Research Center, Materials Technologies, TÜBİTAK Gebze Campus, 41470 Gebze, Kocaeli Turkey

**Keywords:** Erdafitinib, Electrochemical sensor, Modified electrode, Molten salts, Porous carbon, Voltammetry

## Abstract

The successful fabrication is reported of highly crystalline Co nanoparticles interconnected with zeolitic imidazolate framework (ZIF-12) -based amorphous porous carbon using the molten-salt-assisted approach utilizing NaCl. Single crystal diffractometers (XRD), and X-ray photoelectron spectroscopy (XPS) analyses confirm the codoped amorphous carbon structure. Crystallite size was calculated by Scherrer (34 nm) and Williamson-Hall models (42 nm). The magnetic properties of NPCS (N-doped porous carbon sheet) were studied using a vibrating sample magnetometer (VSM). The NPCS has a magnetic saturation (Ms) value of 1.85 emu/g. Scanning electron microscopy (SEM) and transmission electron microscopy (TEM) analyses show that Co/Co_3_O_4_ nanoparticles are homogeneously distributed in the carbon matrix. While a low melting point eutectic salt acts as an ionic liquid solvent, ZIF-12, at high temperature, leading cobalt nanoparticles with a trace amount of Co_3_O_4_ interconnected by conductive amorphous carbon. In addition, the surface area (89.04 m^2^/g) and pore architectures of amorphous carbon embedded with Co nanoparticles are created using the molten salt approach. Thanks to this inexpensive and effective method, the optimal composite porous carbon structures were obtained with the strategy using NaCl salt and showed distinct electrochemical performance on electrochemical methodology revealing the analytical profile of Erdatifinib (ERD) as a sensor modifier. The linear response spanned from 0.01 to 7.38 μM, featuring a limit of detection (LOD) of 3.36 nM and a limit of quantification (LOQ) of 11.2 nM. The developed sensor was examined in terms of selectivity, repeatability, and reproducibility. The fabricated electrode was utilized for the quantification of Erdafitinib in urine samples and pharmaceutical dosage forms. This research provides a fresh outlook on the advancements in electrochemical sensor technology concerning the development and detection of anticancer drugs within the realms of medicine and pharmacology.

**Graphical Abstract:**

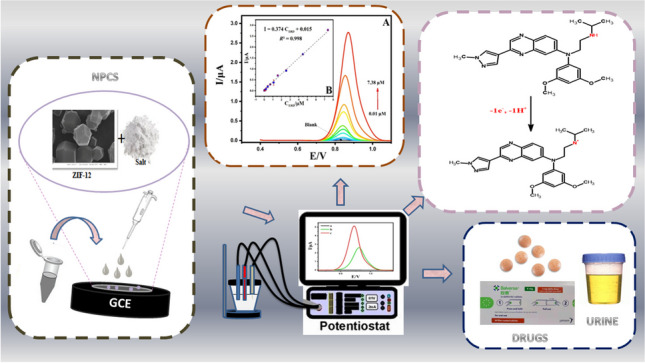

**Supplementary Information:**

The online version contains supplementary material available at 10.1007/s00604-024-06318-z.

## Introduction

Cancer, which is known to have various, is one of the most common causes of mortality globally [1]. Bladder cancer, with urothelial carcinoma being its predominant subtype, is a highly prevalent malignancy on a global scale, holding the 10th position in terms of cancer incidence worldwide. It affects men more commonly than women and is characterized by elevated levels of morbidity and mortality [2–4]. This underscores the significant impact of bladder cancer on public health, emphasizing its substantial role in the landscape of oncological diseases. The pathogenesis of various malignancies, including urothelial carcinoma, has been intricately linked to anomalous fibroblast growth factor receptors (FGFR) signaling pathways [5]. FGFRs, pivotal players in cellular processes such as proliferation, differentiation, and growth, also regulate cell migration and the selective induction of apoptosis during embryogenesis and angiogenesis [6]. This intricate orchestration makes FGFRs attractive targets for anti-neoplastic pharmaceutical agents. Demonstrated inhibitory effects on cellular proliferation and the induction of programmed cell death across diverse tumor models bearing FGFR aberrations have led numerous research consortia to embrace FGFRs as prime targets for therapeutic development [7]. Erdafitinib is a highly potent and discerning oral pan-FGFR tyrosine kinase inhibitor. This pharmaceutical agent, a product of collaborative research by Astex and Janssen, achieves the distinction of being the first FGFR inhibitor to gain FDA approval in 2019 for utilization in the treatment of metastatic urothelial carcinoma (UC) [8]. Erdafitinib exerts its action by impeding FGFR phosphorylation, thus curtailing FGFR-mediated signal transduction cascades. This intervention serves to preclude tumor cell proliferation and provoke programmed cell death, thereby contributing to its anti-neoplastic effect [9].

The compound’s IUPAC nomenclature is denoted as N’-(3,5-dimethoxyphenyl)-N’-[3-(1-methylpyrazol-4-yl)quinoxalin-6-yl]-N-propan-2-ylethane-1,2-diamine (Figure S1) [10]. Erdafitinib is classified within diverse pharmacological categories, such as anti-neoplastics, pyrazoles, diamines, and quinoxalines. This comprehensive categorization is crucial in characterizing Erdafitinib within the broader pharmacological landscape, elucidating its molecular and structural attributes that contribute to its pharmacotherapeutic profile. Such precise delineation facilitates a nuanced understanding of Erdafitinib’s pharmacological identity, providing a foundation for its contextualization and exploration within the intricate domain of pharmaceutical sciences [11].

Beyond all these, the active ingredient Erdafitinib causes many negative effects on human health, such as hyperphosphatemia, hyponatremia, stomatitis, asthenia, nail dystrophy, urinary tract infection, and palmar-plantar erythrodysesthesia syndrome. Therefore, the determination of Erdafitinib is of great importance to prevent side effects, especially considering the high reported rates of ocular toxicity [12–14].

Examination of the existing literature pertaining to Erdafitinib (ERD) disclosed a paucity of documented analytical methodologies available for the quantitative determination of this compound. In reported studies, HPLC–UV [15, 16], LC–MS/MS [17], UPLC-MS/MS [18, 19], and spectrofluorimetric methods [20] have been investigated. To our most current knowledge, there is an absence of any established electrochemical methodology documented for the quantification of this compound. Therefore, this investigation distinguishes itself as the inaugural electrochemical analysis conducted on ERD. Additionally, the methods described in the literature exhibit drawbacks such as elevated costs, intricate and time-consuming experimental procedures, and the utilization of large quantities of toxic and hazardous solvents [21–24]. For example, the derivatization steps can extend the analysis time, and certain situations may necessitate the use of purely organic solvents. Consequently, employing the mentioned techniques for analysis would be labor-intensive, requiring derivatization and pre-concentration steps, encompassing sampling and various forms of extraction before conducting the actual analyses [25]. Thus, the requirement arises for advanced laboratories and skilled manpower [26]. The LC–MS/MS provides enhanced specificity and sensitivity; however, its primary drawbacks include the substantial instrument costs and limited availability in various centers [27]. HPLC–UV is favored due to its greater accessibility compared to the expensive alternative method. However, it has a lengthier run time and demands a larger sample volume owing to its lower sensitivity [28]. Besides all this, Electrochemical methods stand out as alternatives among various detection techniques because of their cost-effectiveness, prompt response, high sensitivity, and selectivity for drug analysis molecular detection, coupled with reduced reagent consumption [29, 30]. Furthermore, electrochemical sensors are considered to be more selective, efficient and sensitive compared to alternative methods, primarily due to their ability to improve through various modifiers [31–34].

Carbon materials such as graphene, graphite, and activated carbon have been well-known due to their unique and important characteristics, such as large specific surface area, abundant resources, high chemical resistance, and high electronic conductivity [35, 36]. After KOH activation, the first superactive carbon with an extremely large surface area was formed [37, 38]. However, this material is quite expensive due to the large amount of alkali utilized in its production. For the production of activated carbon, various inorganic salt derivatives have been used to create superactive carbon [39, 40]. Recently, a simple and sustainable method for the synthesis of extremely porous functional carbons, known as “salting templating” [41], has been disclosed. At increased temperatures, a carbon precursor is combined with a non-carbonizable inorganic salt, which is then carbonized and scaffolded. This method yields carbonized networks that maintain the structure of their inorganic counterparts while keeping their exceptional porosity and pore size. The molten salt synthesis process is a cost-effective and efficient way to produce carbon-based products with excellent yields [42, 43]. By utilizing molten salts as a liquid reaction media and pre-formed templates, this approach is frequently utilized to manufacture diverse carbon nanostructures using a variety of carbon precursors and inorganic salts [44–46]. Metal–organic frameworks (MOFs) are a novel type of porous materials that combine organic and inorganic components [47–49]. The distinct advantages of MOFs, such as their crystalline porous structure, highly dispersed metal components, and adjustable pore size, have led to their extensive investigation in gas storage [50], separation [51], purification [52], catalysis [53–57], drug delivery [58–60], sensing [61], thin-film systems [62, 63], energy storage devices [64], and for the fabricate a conductive composite porous amorphous carbon [48, 65]. All depends on the characteristics and ultimate structures,various synthetic methods can be used to generate MOFs, each with its own set of advantages and disadvantages. Slow diffusion [66], hydrothermal (solvothermal) [67], electrochemical [68], mechanochemical [69], microwave-assisted heating, and ultrasound [70] are some of the synthesis methods frequently used in the synthesis of MOFs. There are a few drawbacks to MOFs despite their many benefits; for example, they have a low quantum yield in luminescence chemical sensing, and electrochemistry suffers from limited charge transfer and significant charge recombination. However, these drawbacks can be overcome by creating composite materials based on MOFs. Carbons formed from MOFs via direct pyrolysis frequently exhibit architectures dominated by micropores, which significantly retard reaction kinetics by limiting mass transfer and providing access to active areas within micropores. To enhance the application potential of MOF-derived carbons in Lithium-ion batteries (LIBs), recent studies have focused on preparing hierarchical porous carbons from MOFs [71]. Most prior papers on carbons generated from MOFs sought to create porous carbons by direct pyrolysis [72]. As a result of the inescapable formation of additional C–C or C-N bonds between adjacent MOF particles, high-temperature annealing induces an irreversible fusion/aggregation of nanoparticles and a partial morphological collapse of MOFs [48]. Among these strategies, nanostructuring and hybridization with conductive materials, such as carbon, have become reliable and prominent methods [73, 74]. A material containing nanosized metal and carbon particles is very effective because nanosized carbon may not just alleviate the strain induced by the volume expansion of nanosized metals and alloys but also enhance a material’s conductivity to facilitate rapid charge and ion transfer. Diverse carbon materials, including as carbon nanotubes [75], graphene [76, 77], and amorphous carbon [78] have been employed in this context to produce carbon composites/ metals/ metal oxides/ and alloys with significantly enhanced electrochemical performance [79, 80].

The objective of this research is to intensify the production of a conductive carbon at maximum surface area and product yield from ZIF-12 by applying the pyrolysis of ZIF-12 with NaCl. We report a simple molten salt-assisted method for preparing Co nanoparticle-embedded interconnected porous carbon structures. During the pyrolysis, the NaCl salt is used and activates the surface of ZIF-12 particles and connects them into carbon skeletons. The salt crystal functions as a restricted reactor for the degradation of organic intermediates, which then generate graphene-like carbon nanosheets during the carbonization process. ZIF-12 provides the carbon and nitrogen necessary for the creation of an N-doped porous carbon sheet (NPCS). The prepared amorphous NPCS is N-doped and defect-rich. The 3D macroscopic structure encourages mass diffusion, while the nanosheets connecting it encourage electrical conductivity. The obtained interconnected carbon skeletons generated after NaCl removal become macropores, facilitating rapid electron/ion transport pathways that enhance the kinetics of the process.

The main contributions presented in this paper include the following: (1) synthesis of NPCS by calcining NaCl-doped ZIF-12; (2) determination of crystal size, surface area, and morphology of NPCS by XRD, XPS, and SEM-TEM; (3) determination of Erdafitinib in synthetic human urine samples and pharmaceutical dosage forms using an electrochemical method. On the other hand, discovering new anticancer drugs and screening their efficacy, avoiding possible adverse effects, require a huge amount of resources and time-consuming processes. Streamlining the time and resources involved in this procedure also plays a crucial role in advancing the development of novel anticancer drugs [81]. Moreover, in clinical application, this approach could successfully enhance the efficacy and safety of chemotherapy regimens [82]. Consequently, the first electrochemical sensor capable of accurately measuring amounts of Erdafitinib was created using a modified electrode with NPCS. The porous MOF-derived amorphous carbon composite NPCS could exhibit a high and stable performance in the detection of Erdafitinib. This study sheds light on the effective structural design and fabrication strategy for the highly efficient analytical profile of Erdafitinib via the modification of electrodes with a NPCS material which has interconnected and macroporous features. To this end, a simple and low-cost process is developed to produce hierarchical porous amorphous carbon, which could be potentially used in sensors and detecting devices.

The objective of this study is to investigate and clarify the possible oxidation mechanism of ERD on NPCS/GCE using CV and DPV. Also, another main purpose is to establish a meticulously validated electrochemical methodology designed for the quantification of Erdafitinib within pharmaceutical dosage formulations and synthetic human urine specimens.

## Experimental section

### Reagents and apparatus

For a more comprehensive understanding and detailed insights, it is recommended that readers refer to the Supplementary Information.

### Synthesis of ZIF-12

Zeolite imidazolate frameworks (ZIF-12) were synthesized according to the literature with slight modifications [83]. Briefly, 2 mmol of benzimidazole with NH_3_ (1 mmol) and 1 mmol of cobalt acetate tetrahydrate ((CH_3_COO)_2_Co.4H_2_O) was dispersed into two 10 mL of methanol-toluene (3:1 mol%) solution. The two suspensions were then combined and agitated briskly at room temperature for three hours, and purple powders were centrifuged with methanol. An overnight vacuum drying process at 60 °C produced the final product, which manifested as a purple powder.

### Preparation of N-doped porous carbon sheets

One gram of ZIF-12 was introduced to five grams of a supersaturated salt solution while vigorously stirring for more than 24 h. Once the salt had completely recrystallized, the temperature was gradually increased to 80 °C in a water bath. Following ten hours of vacuum drying at 60 °C, the final product was achieved by subjecting the dried powder to Ar gas at a rate of 2 °C min^−1^ at 800 °C for three hours. The product was acquired by subjecting it to a series of processes, beginning with washing with deionized water, filtration, and drying at 120 °C. The products were obtained and named N-doped porous carbon sheets (NPCS).

### Preparation of the modified glassy carbon electrode

Initially, the unmodified Glassy Carbon Electrode (GCE) underwent a meticulous cleansing procedure following a previously documented protocol [84]. The meticulously cleaned electrode was then subjected to controlled drying for 10 min at room temperature (~ 24 °C). Subsequently, a precisely measured volume of 6.0 μL of a homogenized suspension containing NPCS composites at a concentration of 0.5 mg/mL, dissolved in deionized water, was meticulously drop-casted onto the impeccably smooth surface of GCE. Following this deposition, the modified electrode, referred to as NPCS/GCE, underwent a natural drying process at ambient room temperature. Once the solvent had completely evaporated, the NPCS/GCE electrode was then immersed within an electrochemical cell to facilitate a series of electrochemical tests [85].

### Preparation of human synthetic urine samples and dosage forms

The electrochemical detection of ERD was rigorously assessed in actual samples, encompassing pharmaceutical tablets and synthetic human urine specimens. Five tablets of BALVERSA®, each containing 4.0 mg of the active ingredient, were meticulously weighed and subsequently subjected to homogenization. The mean tablet weight was accurately determined through a rigorous calculation process. Furthermore, for the preparation of a 1.0 mM tablet stock solution, a specific quantity of this homogenized powder was meticulously extracted from the mixture, followed by dispersion in deionized water and methanol (1:1). This resulting dispersion was then subjected to a 30-min ultrasonic bath treatment. The resultant solution was further refined by passing it via a 0.45 μm polytetrafluoroethylene (PTFE) filter, then ultimately diluted in the appropriate buffer solution [86]. Synthetic human urine was used as received. The differential pulse voltammetry (DPV) method was judiciously utilized to analyze the synthetic human urine samples, which were intentionally augmented with varying concentrations of ERD [87].

## Results and discussion

### Synthesis and characterization of NPCS material

**Fig. 1:**
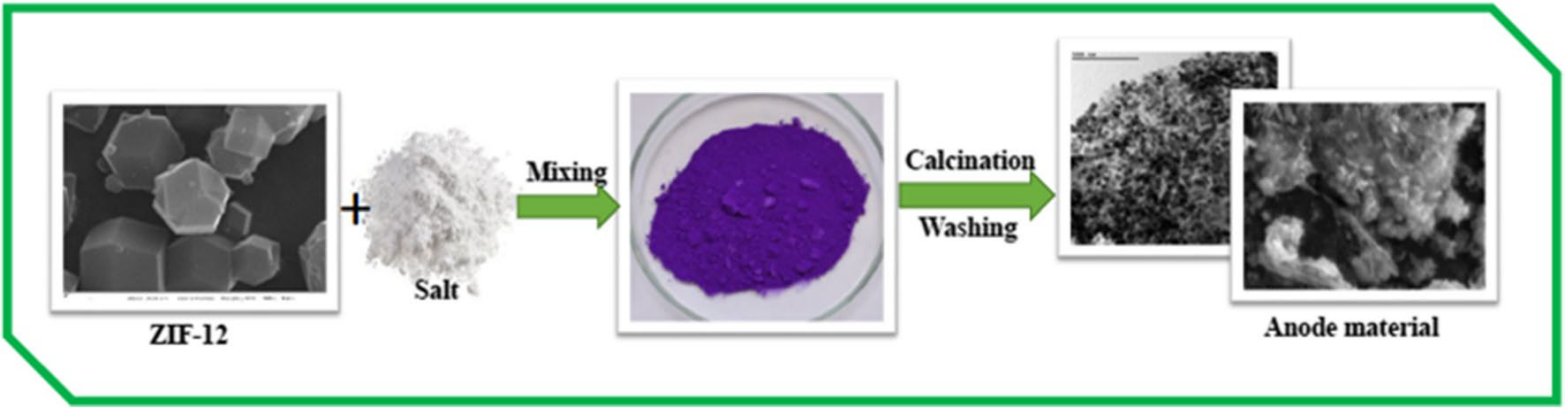
Summary illustration of the synthesis procedures for NPCS

A simplified diagram of the molten-salt-assisted process for synthesizing NPCS material is shown in Fig. 1. A description of the formation of the NPCS product follows: ZIF-12 doped with NaCl is calcined in an Ar atmosphere. When heated to the molten point of NaCl, the molten salts induce ionized species reactions between cobalt salts, resulting in the growth of nanoparticle cobalt and a trace amount of cobalt oxide crystals under the influence of the molten salts’ “molecular template.”

**Fig. 2:**
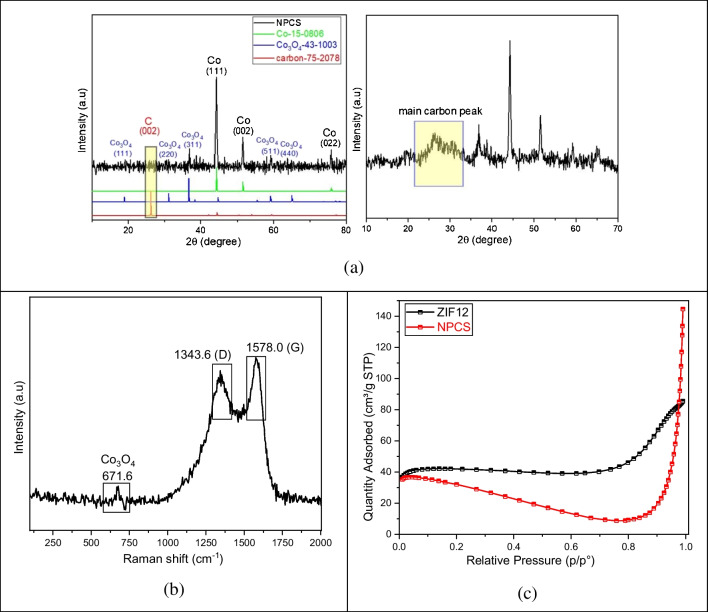
XRD analysis (**a**), Raman analysis (**b**), nitrogen adsorption isotherm of NPCS (**c**)

The X-ray diffraction (XRD) patterns of NPCS are given in Fig. 2a. The sample matches well with the cubic (Fm-3 m, 225) Co (PDF#15–0806) with the diffraction peaks of (111), (002), and (022) crystalline planes and a trace amount of Co_3_O_4_ crystals (PDF#43–1003). The peak of carbon (PDF#75–2078) was also detected in the NPCS sample. The carbon diffraction peak is too weak to be properly detected. The presence of a broad peak at around 2θ = 26.6° for the carbon powder suggests that the carbon in the Co/C nanocomposites is mainly amorphous, as opposed to the characteristic sharp peak of graphite carbon at 2θ = 26.6°. Nevertheless, the carbon content of NPCS material could be determined via XPS analysis. The carbon concentration of the NPCS sample was 75.86%, as shown in Table 1. The crystallite size D can be calculated from the XRD via the Debye-Scherer formula,

$$D=\frac{0.9\lambda }{\beta cos\theta }$$

where*β* is the full width of half maximum in (2*θ*), *θ* is the corresponding Bragg angle, λ = 0.154 nm. The crystalite size for Co NP is calculated with reference to the maximum peak at angle at ~ 44°. The average crystallite size for Co NP is found to be ~ 35 nm.

**Table 1:** Elemental contents of ZIF-12 and NPCSmaterials according to XPS analysis

Sample	C%	Co%	BET surface area (m^2^/g)
ZIF-12	80.99	19.01	128.6
NPCS	75.86	23.35	89.04

Williamson-Hall method was performed in order to calculate the average crystallite size (D) and the best agreement between the experimental and fitted data with R^2^ = 0.94 (D ≈ 42 nm) (Figure S2).The crystallite size D can be calculated from the XRD via the Williamson-Hall method formula,

$$\beta {\text{cos}}\left(\theta \right)=\frac{k\lambda }{D}+\eta {\text{sin}}(\theta )$$

Scherrer formula ( k, λ, β, and θ are the shape factor taken as 0.9, the X-ray wavelength, the full width at half maximum (FWHM), and the Bragg angle of the peak, respectively) considers only the effect of crystallite size on the XRD peak broadening. By the Scherrer equation, the average crystallite size calculated from the intercept of the obtained fitted line is 34 nm.

D = Kλ/βcosθ.

When we compare these two methods, the Williamson-Hall approach gives larger apparent crystallite sizes than the Scherrer equation due to its consideration of lattice strain effects, as well as the additional complexity involved in its calculations.

The above XRD and SEM–EDS analysis indicates that cobalt in the NaCl-doped ZIF-12 has been transformed to metallic Co and a trace amount of Co_3_O_4_ crystals by pyrolysis. In addition, Raman analysis confirms this observation (Fig. 2b). Consequently, we might hypothesize that during calcination, the molten salt form a liquid reaction environment in which the reactants are easily able to interact and clash with one another. The nitrogen gas absorption curves (Fig. 2c) provide additional evidence that the melting salt and evaporation process can significantly reduce the specific surface area of the NPCS.

Specially, the BET surface areas of the ZIF-12 and NPCS are shown in Table 1. According to Table 1, the ZIF-12 has a slightly bigger surface area than NPCS. This could perhaps be attributed to the pristine crystal structure of ZIF-12 collapsing, as seen by the microporous nature of the NPCS structure as revealed by SEM and TEM images. The evaluation of the N_2_ adsorption isotherm reveals that NPCS possesses type I isotherms, which provide confirmation of its microporous pore structure.

**Fig. 3:**
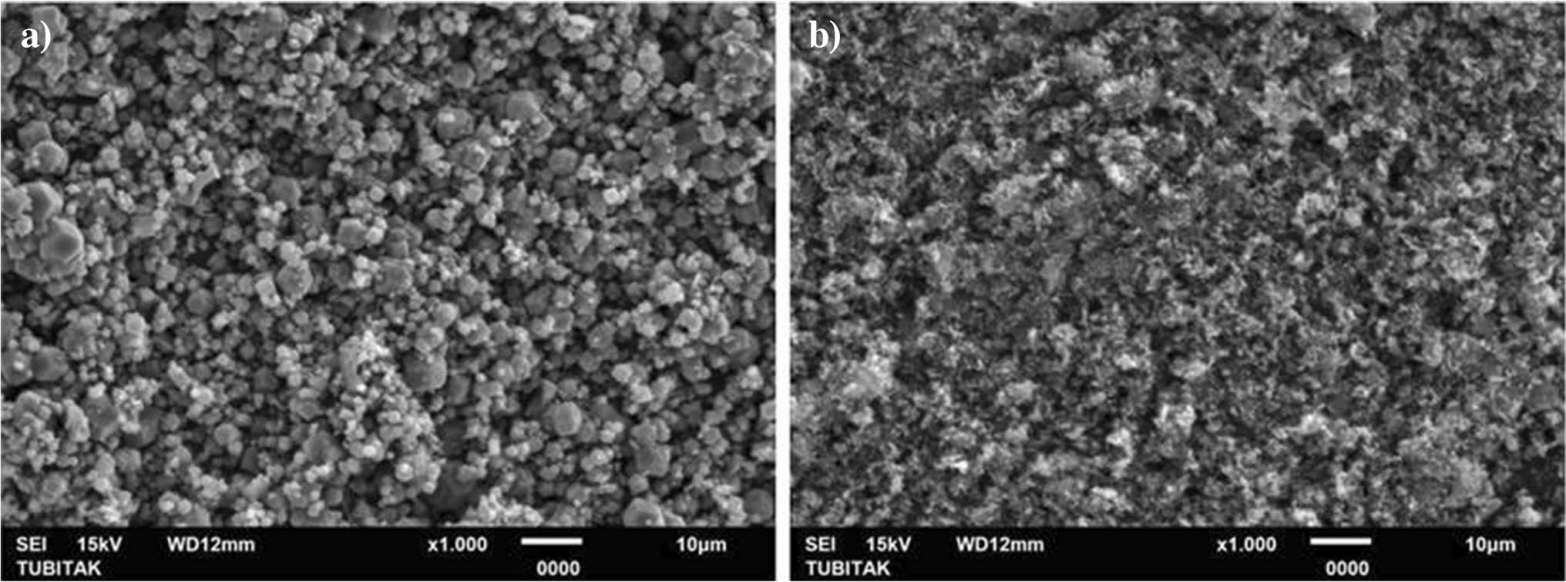
SEM images of ZIF-12 (**a**) and NPCS (**b**) specimens

**Fig. 4:**
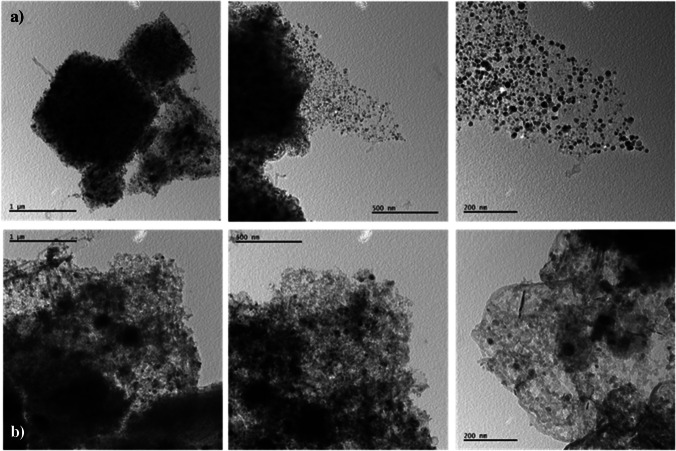
TEM images of ZIF-12 (**a**) and NPCS (**b**) specimens

The scanning electron microscopy (SEM) and transmission electron microscopy (TEM) images of ZIF-12 and NPCS specimens are shown in Fig. 3 and Fig. 4, respectively. The morphologies of ZIF-12 and NPCS are notably dissimilar, as the salt-activated structure of the latter is highly dependent on the salt employed during carbonization. The NPCS synthesized exhibits a more integrated morphology with irregular pits on its surfaces.

The elemental mapping outcomes of SEM-energy dispersive spectroscopy (EDS) (Figure S3) indicate that the C, O, and Co elements are uniformly distributed across the entire area of the amorphous carbon network. It is noteworthy to mention that the trace levels of N signal, which align with the XPS findings, primarily derive from nitrogen-containing heterocyclic compounds inherent in ZIF-12. These heteroatoms may promote electron transport and perhaps generate further defects [88].

**Fig. 5:**
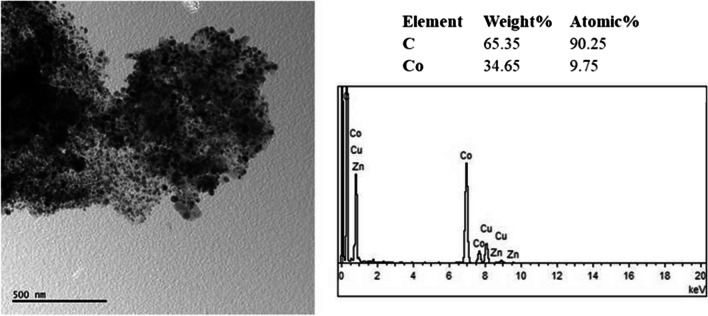
TEM image, EDS spectrum, and EDS result of ZIF-12 sample

**Fig. 6:**
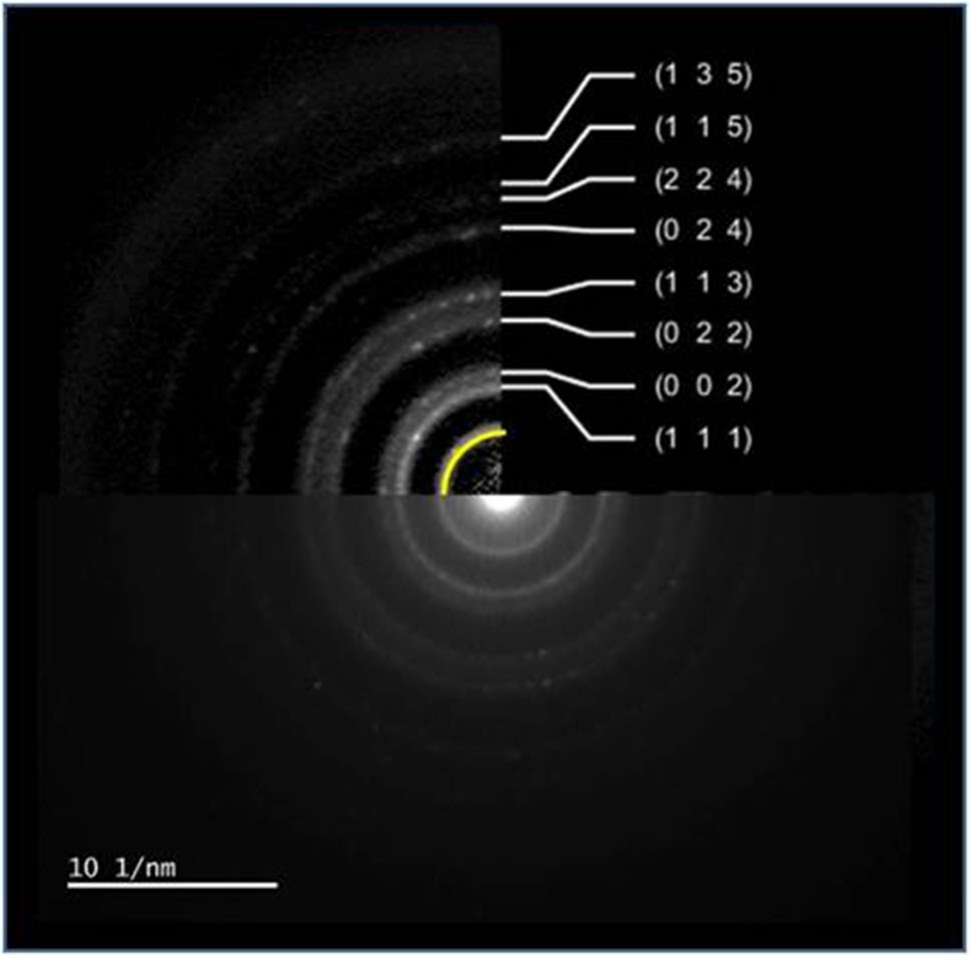
Selected area electron diffraction (SAED) pattern and indexation results

Figure 5 shows the TEM image, EDS spectrum, and EDS result of the ZIF-12 sample. It is observed that the nanoparticles are cobalt based. No oxide phase is seen since EDS did not show any oxygen element. Similarly selected area electron diffraction (SAED) pattern of this image gave only the cobalt phase in addition to carbon (Fig. 6). Cobalt diffraction rings were spotty. A faint dispersed diffraction ring of carbon is evidence of amorphous carbon. Indexation according to Fm-3 m cobalt is also shown in Fig. 6.

**Fig. 7:**
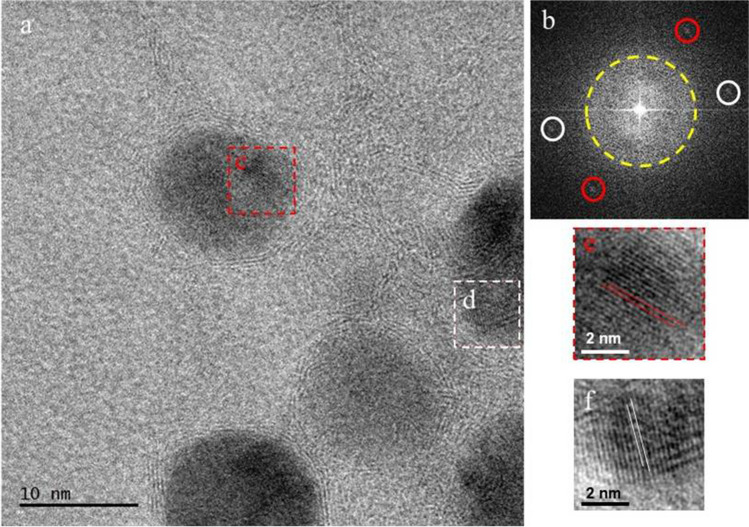
HRTEM image (a) and its fast Fourier transformation (FFT) diffractogram of ZIF-12 sample. HRTEM images labeled (e) and (f) are magnified views of (c) and (d), respectively showing (111) d-spacings of cobalt

In Fig. 7, HRTEM image (a) and its fast Fourier transformation (FFT) diffractogram of the ZIF-12 sample are given. HRTEM images labeled (e) and (f) are magnified views of (c) and (d), respectively showing (111) d-spacings of cobalt. d-spacings measured from both FFT diffractogram and atomic lattice images are 0.205 nm, which matches with (111) d-spacing of cobalt. In the image, lattices labeled with red lines in (e) are parallel to FFT spots labeled with red circles in (b); similarly, lattices labeled with white lines in (f) are parallel to FFT spots labeled with white circles in (b). The yellow-spotted line in FFT guides the eye to the diffused ring pattern of the carbon lattice and also the amorphous carbon background.

**Fig. 8:**
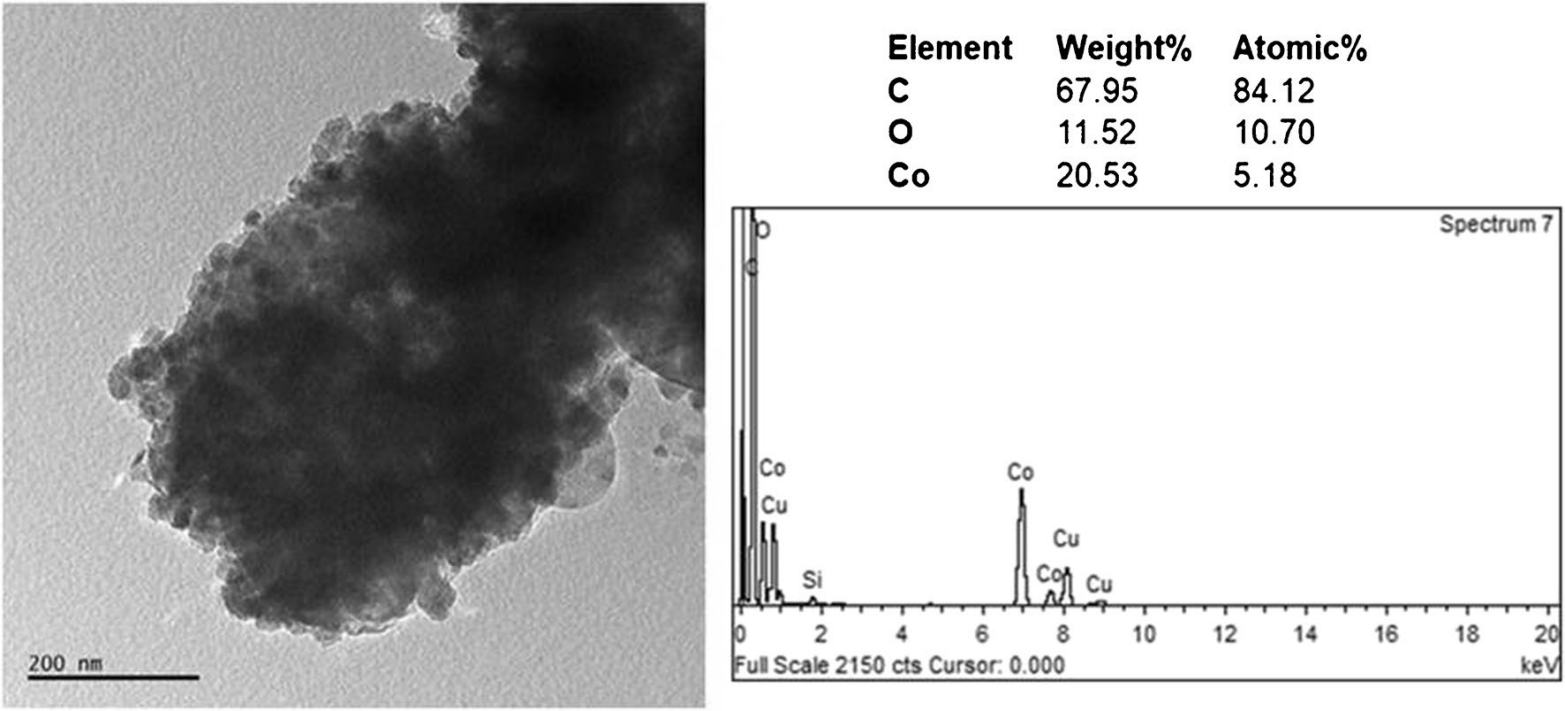
TEM image, EDS spectrum, and EDS result of Co_3_O_4_ nanoparticles in NPCS sample

**Fig. 9:**
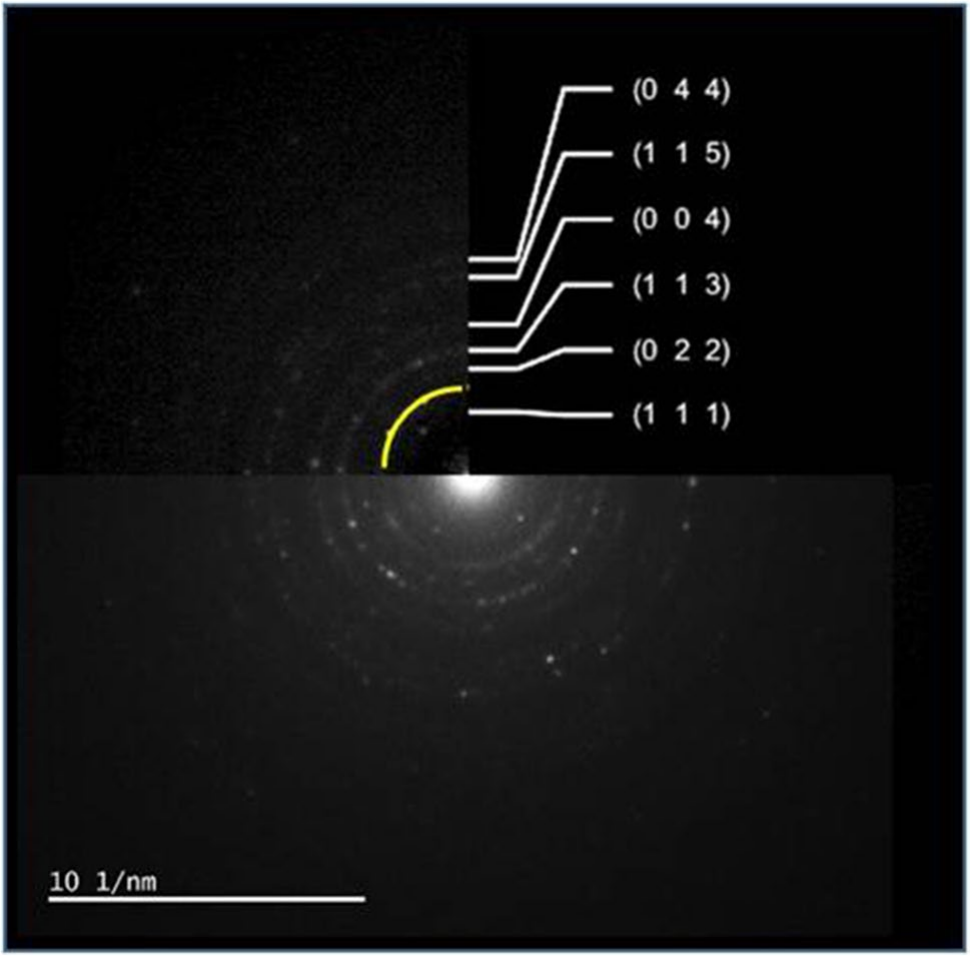
Selected area electron diffraction (SAED) pattern and indexation results of Co_3_O_4_ nanoparticles in NPCS sample

Even if it is rare in some regions of the NPCS sample, Co_3_O_4_ nanoparticles were observed via TEM studies, one representative TEM image, EDS spectrum, and EDS result of Co_3_O_4_ nanoparticles in the NPCS sample given in Fig. 8. In contrast to cobalt nanoparticles, evidence of oxygen in TEM-EDS results is the first proof of Co_3_O_4_ nanoparticles. The second and direct evidence of nanoparticles being in the Co_3_O_4_ phase is the selected area electron diffraction results. In Fig. 9, we indexed the ring patterns according to the Co_3_O_4_ phase. Moreover, the yellow-labeled diffuse ring comes from the carbon. Since it is diffused, it can be stated that carbon is amorphous in the NPCS sample, similar to the ZIF-12 sample.

**Fig. 10:**
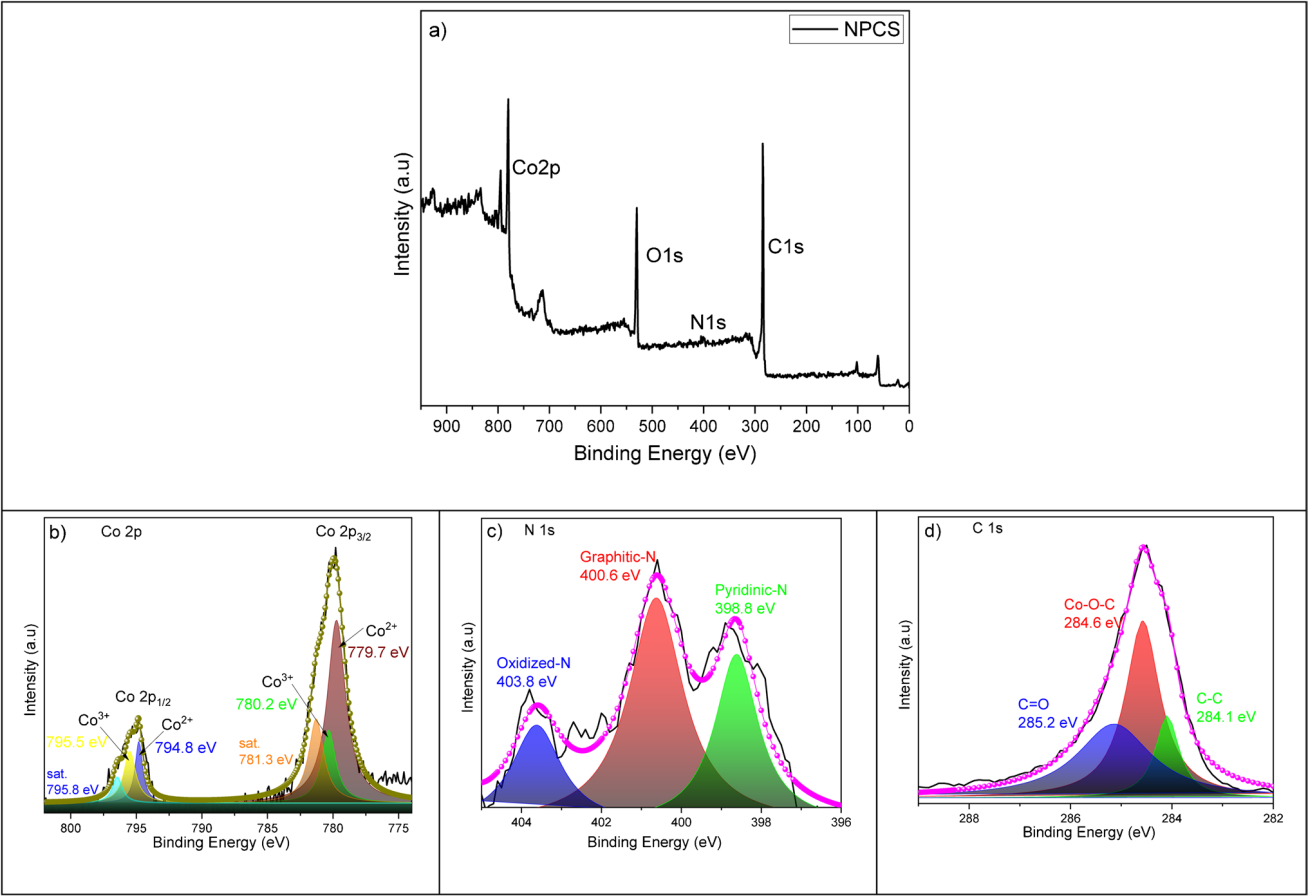
Survey XPS spectra for NPCS, the high-resolution XPS spectra (**a**) of Co 2p (**b**), N 1 s (**c**), C 1 s for NPCS material (**d)**

In the interim, XPS studies are employed to determine the elemental composition of the as-prepared NPCS surface. The identification of the peaks at 780.0, 533.0, 401.0, and 285.0 eV, corresponding to Co 2p, O 1 s, N 1 s, and C 1 s, is illustrated in Fig. 10a. The deconvolution spectra of Co elements are displayed in Fig. 10b, and the peaks at 779.7 eV and 780.2 eV both belong to Co 2p_3/2_; the peaks at 794.8 eV and 795.5 eV belong to Co 2p_1/2_; 781.3 eV and 795.5 eV are two satellite peaks, and these are the characteristic peaks of Co_3_O_4_ phases [89]. The peaks with the binding energy of 779.7 eV and 794.8 eV can be appointed to Co^2+^, while the peaks at 780.2 eV and 795.5 eV can be appointed to Co^3+^. Among them, the two peaks located at 779.7 eV and 794.8 eV originated from Co–C bonds; the peaks located at 780.2 eV and 795.5 eV represent Co–O bonds, which may be due to the surface oxidation of Co atoms during preparation and storage of NPCS material. The total percent of the signal of Co^3+^ and Co^2+^ are relatively high in the total integrated intensity of Co 2p (Co nanoparticle), which is mainly attributed to the limited detection depth of XPS, and thus, the signal of metallic cobalt is not high as much as Co^3+^ and Co^2+^ signals. Considering that Co_3_O_4_ is formed as a result of the oxidation of Co nanoparticles on the surface, XPS analysis is expected. According to XPS results, the formation of Co_3_O_4_ is very low due to the protection of amorphous carbon, which has been demonstrated by the weak intensity of Co_3_O_4_ in XRD spectra. Furthermore, the high-resolution N 1 s spectrum reveals the presence of three distinct forms of nitrogen within the substance: pyridinic nitrogen, graphitic nitrogen, and oxidized nitrogen (Fig. 10c). Based on XPS analysis, the N element content of NPCSs is approximately 1.2% (Table S1). In Fig. 10d, the C 1 s spectrum of the NPCS reveals three peaks at 284.1 eV, 284.6 eV, and 285.2 eV for NPCS, which correspond to C − C, Co–O-C, and C = O, respectively [90]. It illustrates that carbon is predominantly found in C − C and Co–O-C bonds at 284.1 and 284.6 eV, respectively [91, 92].

**Fig. 11:**
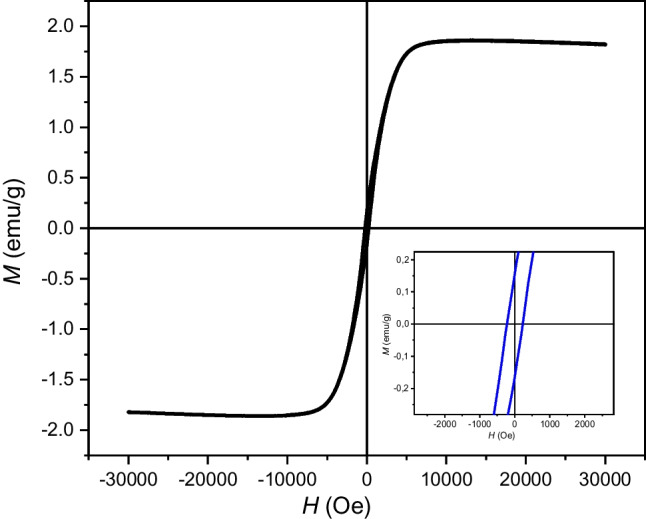
VSM profle of NPCS nanoparticles at 300 K

The magnetic properties of NPCS were studied using quantum design PPMS (Fig. 11), vibrating sample magnetometer (VSM) at 300 K ranging from − 30 to 30 kOe. The magnetic hysteresis loops of NPCS are shown in the figure to access their magnetic properties. The NPCS has a magnetic saturation (Ms) value of 1.85 emu/g. Due to the existence of magnetic metal Co and metal oxide Co_3_O_4_, the sample exhibits ferromagnetic activity and typical S-shaped hysteresis loops under magnetic field excitation.

### The electrochemical characteristics of erdafitinib on both unmodified and modified electrode

The electrochemical attributes of both bare and altered electrodes were examined via the application of differential pulse voltammetry (DPV), cyclic voltammetry (CV) techniques, and electrochemical impedance spectroscopy (EIS).

The electrochemical responses of 10.0 μM Erdafitinib were elicited by employing both an unmodified GCE and an NPCS/GCE. Data acquisition was performed using the DPV technique with the instrumental setting listed in Table 2.

**Table 2:** Instrumental parameters for DPV measurement

Parameter/mode	Setting
Potential ramp	DPV
Start potential	0.4 V
Stop potential	1.2 V
Step potential	5 mV
Scan rate	50 mV/s
Modulation amplitude	50 mV
Modulation time	10 ms

**Fig. 12:**
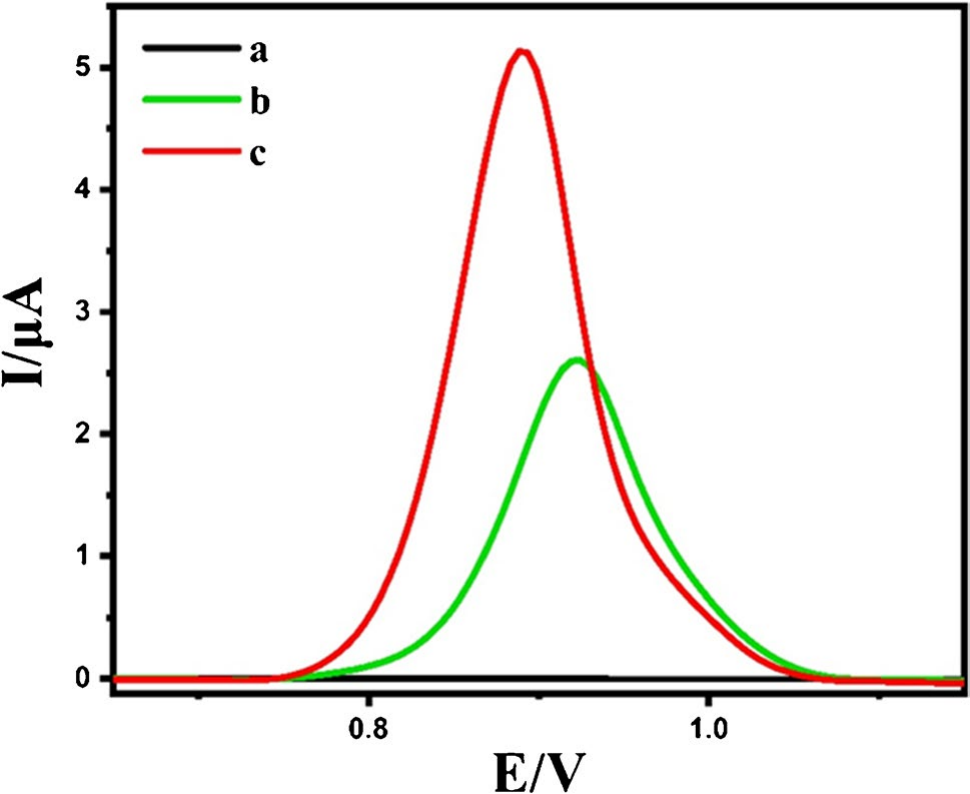
DPVs of 10.0 μM ERD in B-R buffer at pH 6.0 on blank (**a**), unmodified GCE (**b**), and NPCS/GCE (**c**)

The influence of NPCS as an electrode surface modifier is visually represented in Fig. 12. Importantly, the signal of the peak current for Erdafitinib achieved with the newly engineered nanomaterial-modified electrode demonstrated a remarkable ~ twofold amplification in comparison to the unmodified GCE. This enhancement in signal response for Erdafitinib was attributed to the augmented electron transfer kinetics and increased efficient surface area facilitated by the introduction of NPCS nanoparticles on the GCE surface. Moreover, the porosity of the modified electrode can increase the current response over the plain electrode while also causing shifts in peak potentials [93]. Employing chemically modified electrodes offers several advantages, including a reduction in the potential required for the electrochemical reaction to take place and an increase in sensitivity owing to catalytic activity [94].

**Fig. 13:**
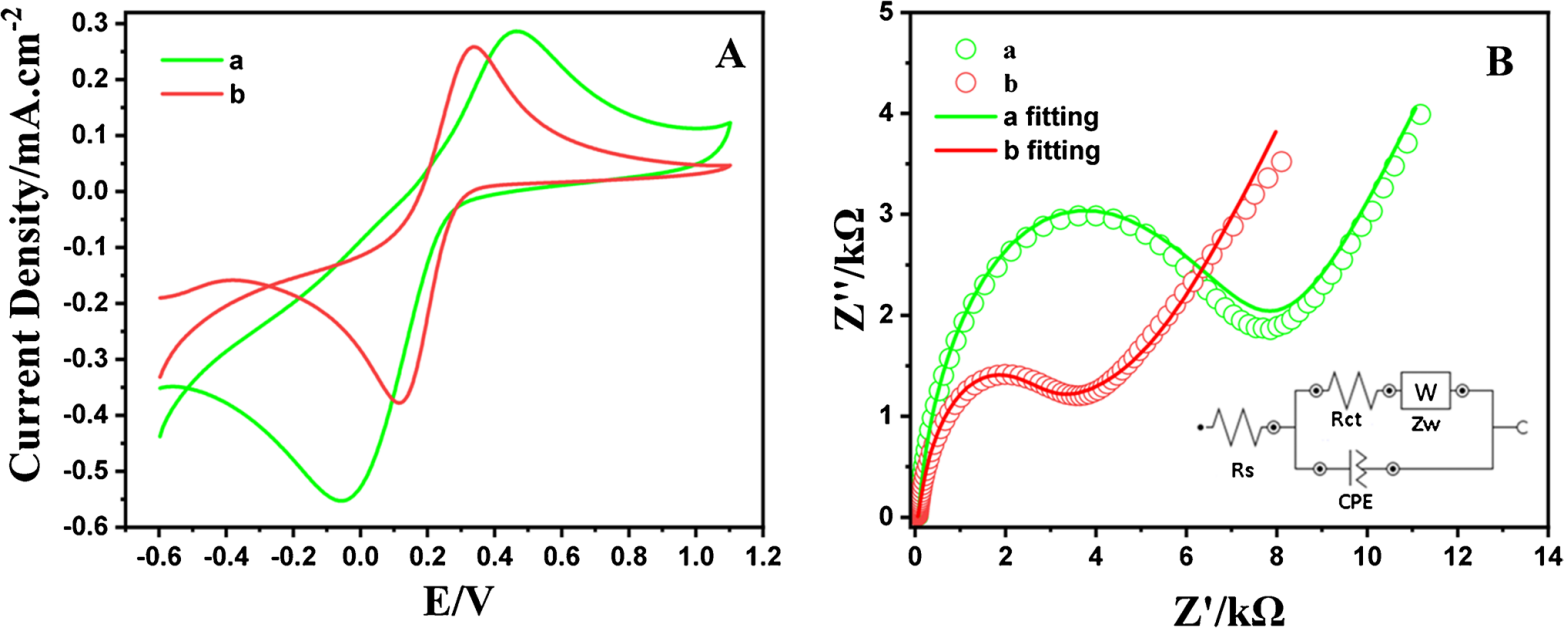
**A** CVs and **B** EIS of the bare GCE (a) and NPCS/GCE (b) in 1.0 mM [Fe(CN)6]^3−/4−^ at a scan rate of 50 mV/s with 0.1 M KCl

The assessment of the electrochemical performance of the fabricated sensor was executed by CV evaluations within a 0.1 M KCl electrolyte solution, using 5.0 mM [K_3_(CN)]_6_^3−/4−^qua the model analyte, and with 50.0 mV/s as the scanning rate (Fig. 13A). The CV profiles ensured detailed information about the electrochemical properties of the distinct electrode configurations. Peak potential separations (ΔEp) were quantified as 0.4760 V and 0.21476 V in the unmodified GCE and NPCS/GCE, severally. The notable reduction in ΔEp monitored for the NPCS/GCE signifies an improved electron transfer rate and a greater potent surface area attributed to the designed electrode. Furthermore, cathodic peak currents along with anodic peak currents also demonstrated remarkable increases, and more distinct peak features were monitored for NPCS/GCE than for the bare GCE. The findings underscore the enhanced electrocatalytic performance of the designed electrode, highlighting its ability to facilitate electrochemical reactions.

EIS emerges as an invaluable technique for the comprehensive investigation of electrode surface conductivity characteristics. Nyquist plots obtained through EIS comprise two discernible segments: a semicircular region and a linear region. The semicircular component is indicative of the charge transfer resistance (Rct) prevalent at higher frequency domains, while the linear segment pertains to lower frequencies associated with diffusional processes [95]. Upon examining the semicircular regions, the Rct observed for the bare GCE was quantified at 7878.9 Ω, which exhibited a notable reduction to 3785.7 Ω after the introduction of functionalized NPCS (as depicted in Fig. 13B). The alterations discerned in the EIS curves can be ascribed to the advantageous attributes of NPCS, which is exceptional electrical conductivity.

The primary factor influencing the sensitivity of the electrochemical sensor is the Electroactive Surface Area (ESA), which dictates the extent of interaction between the analyte and the electrode surface. Therefore, the determination of ESA for both the unmodified and the developed electrode is executed employing the Randles–Sevcik equation (Equation S1) as previously reported employing a 0.1 mM Fe(CN)_6_^3−/4−^ redox probe solution [96]. The calculated ESA for NPCS/GCE was determined to be 0.11 cm^2^ (Figure S4B.), a remarkable approximately twofold increase in comparison to the unmodified GCE (0.0618 cm^2^) (Figure S4A.). These outcomes affirm that the engineered NPCS/GCE manifestly boasts an extensive electroactive surface area, affording an augmented array of reactive parts. This amplified surface area plays a pivotal role in promoting proficient electron transition kinetics and, concurrently, forging a highly potent electron-conductive route connecting the electrode surface with the analyte. As a consequence, these enhancements significantly enhance the comprehensive performance of the electrode.

In addition, the electrical characteristics of an electrode can be assessed through the determination of capacitance. Therefore, existing literature presents a diverse range of experimental protocols for capacitance determination, employing either CV or electrochemical EIS [97, 98]. Initially, CV measurements were employed to ascertain the double-layer capacitance (C_DL_). The calculated C_DL_ values for the unmodified electrode and the modified electrode were 0.3344 μF and 0.8022 μF, respectively. After C_DL_ values, expressed as constant phase element (CPE), obtained from EIS analysis were found to be 0.3718 μF and 0.8428 μF for unmodified electrode and NPCS/GCE, respectively. While the obtained results demonstrated similarity in values, the C_DL_ values obtained through EIS were higher in comparison to those acquired through CV.

### The optimization of electrode modification

The NPCS/GCE optimization, encompassing considerations, for example, compound concentration, quantity, and the characteristics of the backing electrolyte, demands prompt and comprehensive examination. This imperative has prompted the meticulous selection of the most suitable buffer solutions as the preliminary step in this optimization endeavor. Various buffers, including Britton-Robinson (B–R) buffer, phosphate buffer saline (PBS), potassium chloride (KCl), sodium hydroxide (NaOH), hydrochloric acid (HCl), and acetate buffer (AC) underwent thorough investigation. The relationship between the potential peak and oxidation current of ERD in the existence of these diverse supporting electrolytes is graphically delineated in Figure S5A. Especially, among the investigated buffers, the Britton–Robinson buffer exhibited the highest current peak, establishing it as the chosen electrolyte for ensuing research of ERD at the recommended electrode. Otherwise, the effect of the concentration of NPCS composite was methodically investigated at values of 0.2 to 2.0 M (Figure S5B). A discernible enhancement in the oxidative mark of the aimed analytes was notably monitored at a concentration of 0.5 M within the NPCS nanocomposite. Consequently, 0.5 M was determined as the ideal concentration level for the design of NPCS-based electrodes for use in subsequent studies.

Moreover, a comprehensive analysis of the impact of NPCS nanocomposite quantity on the electrochemical electrode’s activity and sensitivity was conducted inside the parameters of 2.0–7.0 μL (Figure S5C). The highest oxidation peak current was realized at 6.0 μL of the nanocomposite, as delineated in Figure S5C. Nevertheless, beyond 6.0 μL, a noticeable decline was monitored, presumably attributed to diminished adherence of the altering stratum to the electrode surface. As a result, it was deduced that the ideal circumstances was obtained at a concentration of 0.5 M and a quantity of 6.0 μL of the NPCS nanocomposite, thereby establishing a robust foundation for subsequent analytical applications.

### The impact of pH

**Fig. 14:**
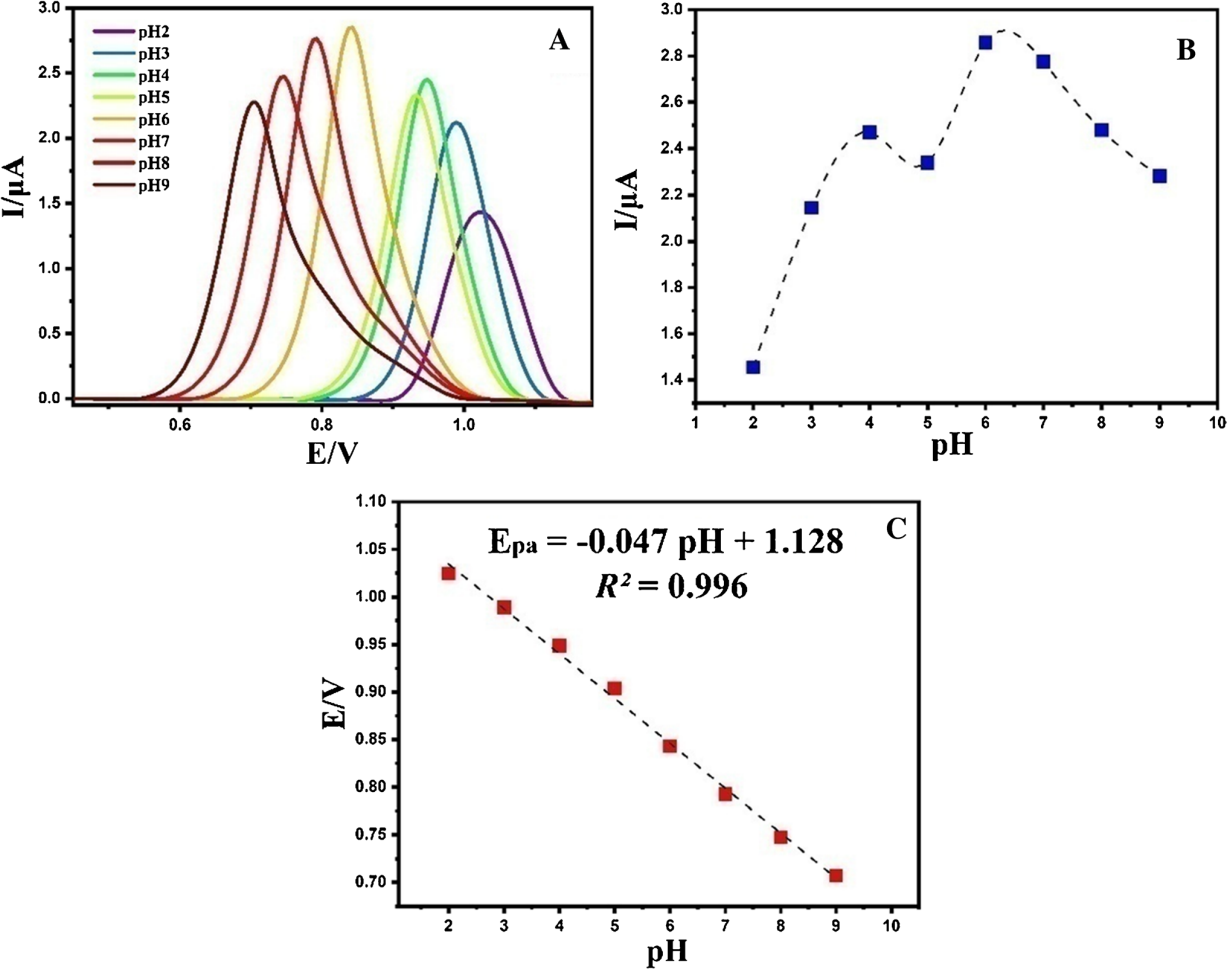
**A** DPV of 10.0 μM of ERD at dissimilar pHs (2.0 to 9.0) on the surface of NPCS/GCE; **B** Impact of pH values on the peak current for developed electrode containing 10.0 μM of ERD at various pHs; and **C** Impact of pH values on the potential for developed electrode containing 10.0 μM of ERD at various pHs

In this research, Britton-Robinson (BR) buffers were deliberately chosen as the electrolyte solution, with a purposeful variation in pH from 2.0 to 9.0. This systematic pH range allowed for a thorough examination of the electrochemical response of the ERD across different protonation states, providing a detailed understanding of its behavior along the acidity-alkalinity spectrum. The recorded profiles from DPV under various pH conditions (Fig. 14A) serve as a meticulous record, capturing subtle changes in peak currents and potentials. A noteworthy finding in our investigation is a distinct increase in peak current at pH 6.0, representing the optimal operational pH where the electrochemical system achieves maximum efficacy and sensitivity. Furthermore, the observed decrease in current amplitude with a further rise in pH is ascribed to the dynamic nature of ERD. (Fig. 14B). Namely, this shift suggests that at low pH, the nitrogen of the ERD molecule is protonated, which makes the loss of an electron more difficult, and at a pH above 6 it is hydrolysis of imine, which leads to a sharp decrease in the ERD oxidation current [99].

The careful examination of the electrochemical behavior of ERD has revealed a noticeable shift in the oxidation peak potential towards more negative values with increasing pH levels (Fig. 14C). This indicates that protons (H^+^) have taken part in the electrochemical oxidation process of ERD [100, 101]. An important outcome of this investigation is the development of a linear regression model expressing the relationship between the oxidation peak potential of ERD and the surrounding pH levels as Epa =  − 0.047pH + 1.128. This model, supported by a high coefficient of determination (*R*^2^ = 0.996), demonstrates exceptional accuracy in capturing the intricate pH-dependency inherent in the electrooxidation of ERD. The key deduction from this complex electrochemical narrative is significant: both electrons and protons play an equitable role in the irreversible oxidation process of ERD at the NPCS/GCE interface. This assertion is reinforced by the observed slope (47 mV/pH) of the relevant oxidation potential curve, which closely approaches the theoretically expected Nernstian slope value of 59.2 mV/pH[102].

### The impact of scan rate

**Fig. 15:**
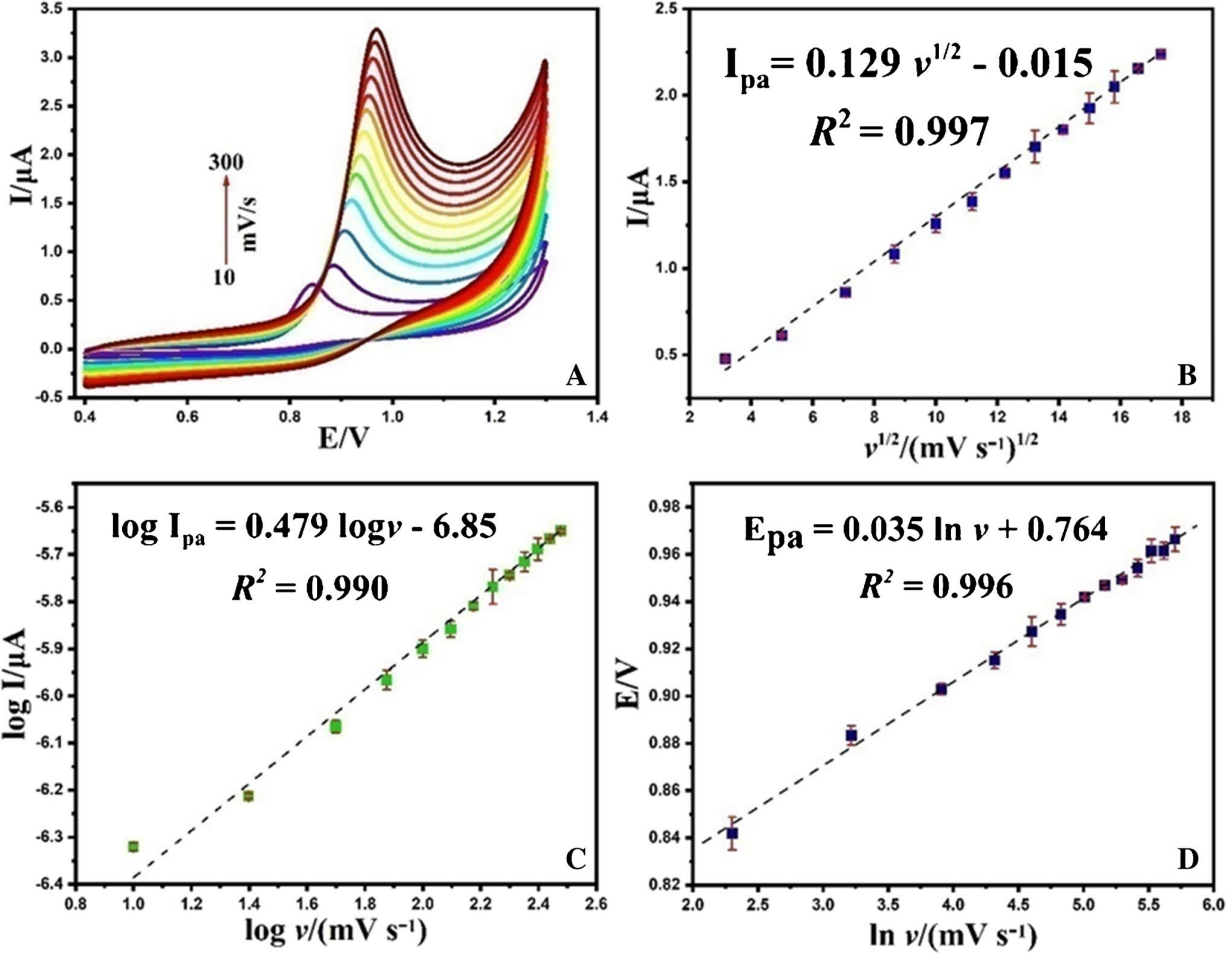
CVs of the ERD (10.0 μM) in NPCS/GCE at dissimilar scanning rates (from 10.0 to 300.0 mV.s^−1^) (**A**); the relevance of Ipa vs. υ.^1/2^ (**B**); the relevance of the log Ipa vs. log υ (**C**); and plot of the Epa vs. ln υ acquired in NPCS/GCE (**D**)

Systematic examination of the scan rate (υ) impact on the voltammetric response of ERD serves as a pivotal step in the elucidation of the intricate electrochemical oxidation mechanism, further enabling the discrimination between a diffusion or adsorption-controlled electrochemical process. In pursuit of this goal, a comprehensive assessment of the electrochemical performance of 10.0 μM ERD on the NPCS/GCE surface, using cyclic voltammetry (CV), was conducted over a scan rate range spanning from 10 to 300 mV/s, all while immersed in a Britton-Robinson (BR) buffer held at a pH of 6.0. The investigation revealed distinctive anodic peaks in the forward scans, with no evident cathodic peaks identified in the backward scans. This observation affirms the irreversible of the oxidation process for ERD (Fig. 15A) [100, 103]. A discernible linear correlation among Ι_pa_ (peak current) and υ^1/2^ (the scan rate square root) was meticulously identified within the range of 10.0 to 300.0 mV/s ( Ι_pa_ = 0.129 υ ^1/2^ – 0.015, R^2^ = 0.997) (Fig. 15B). This observation serves as a robust indicator that the electrochemical reaction is primarily governed by diffusion, thereby elucidating the non-adherence of analyte ions to the electrode surface [100]. Moreover, validation of this diffusion-centric behavior was obtained through the logarithmic representation of the peak current with scan rates, stated by log Ι_pa_ = 0.479 logυ-6.85 (R^2^ = 0.990). Here is the slope of log Ι_pa_ in relation to log υ with between 0.0 and 0.5 (completely 0.479) (Fig. 15C). This suggests that the oxidation of ERD is a diffusion-controlled process [104]. As can be seen in Fig. 15D, a linear connection between E_pa_ and Ιnυ was obtained with an equation of Epa = 0.035 ln v + 0.764, R^2^ = 0.996. Leveraging Leviron’s theory of irreversible electrode reactions (Equation S2) and the slope of the E_pa_versus Ιnυ, were employed to discern the number of electrons exchanged during the electrochemical process [105].

**Scheme 1:**
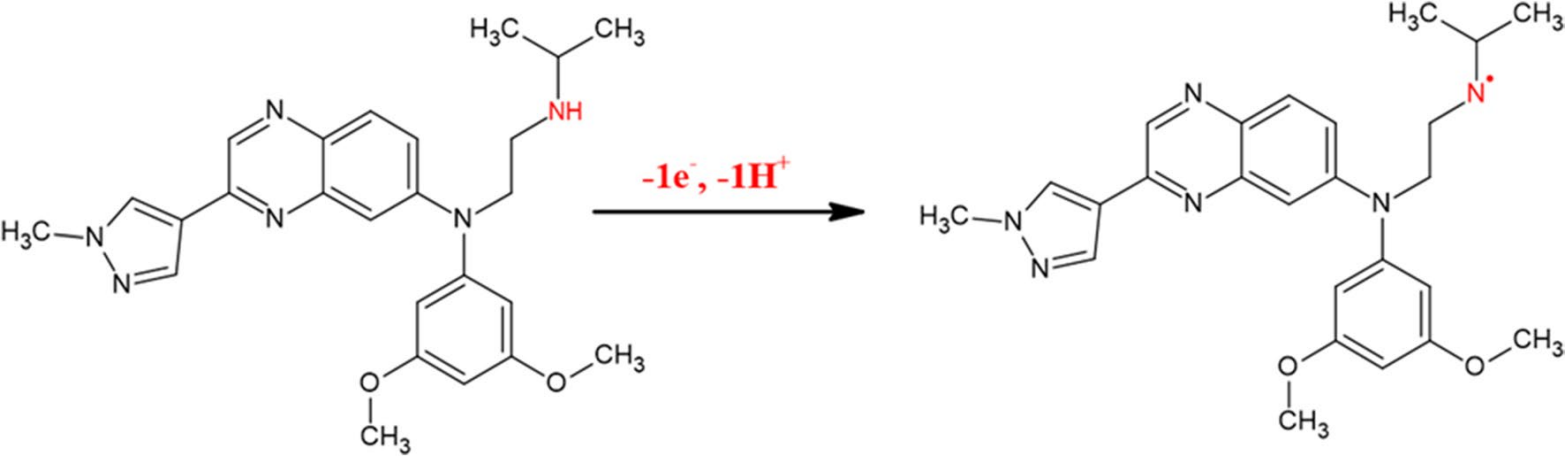
The possible oxidation reaction of ERD

According to estimates, 1.467 (∼1) e^−^ were transported during the electrooxidation reaction of ERD [106]. The findings obtained from this inquiry, combined with those gleaned from the pH scan analysis, provide evidence supporting the requirement for the simultaneous transition of one electron and one proton during the electrochemical oxidation of ERD. The potential oxidation mechanism of ERD on NPCS/GCE is schematized (Scheme 1) [107, 108].

### Analytical performance of the sensor created for the detection of erdafitinib

**Fig. 16:**
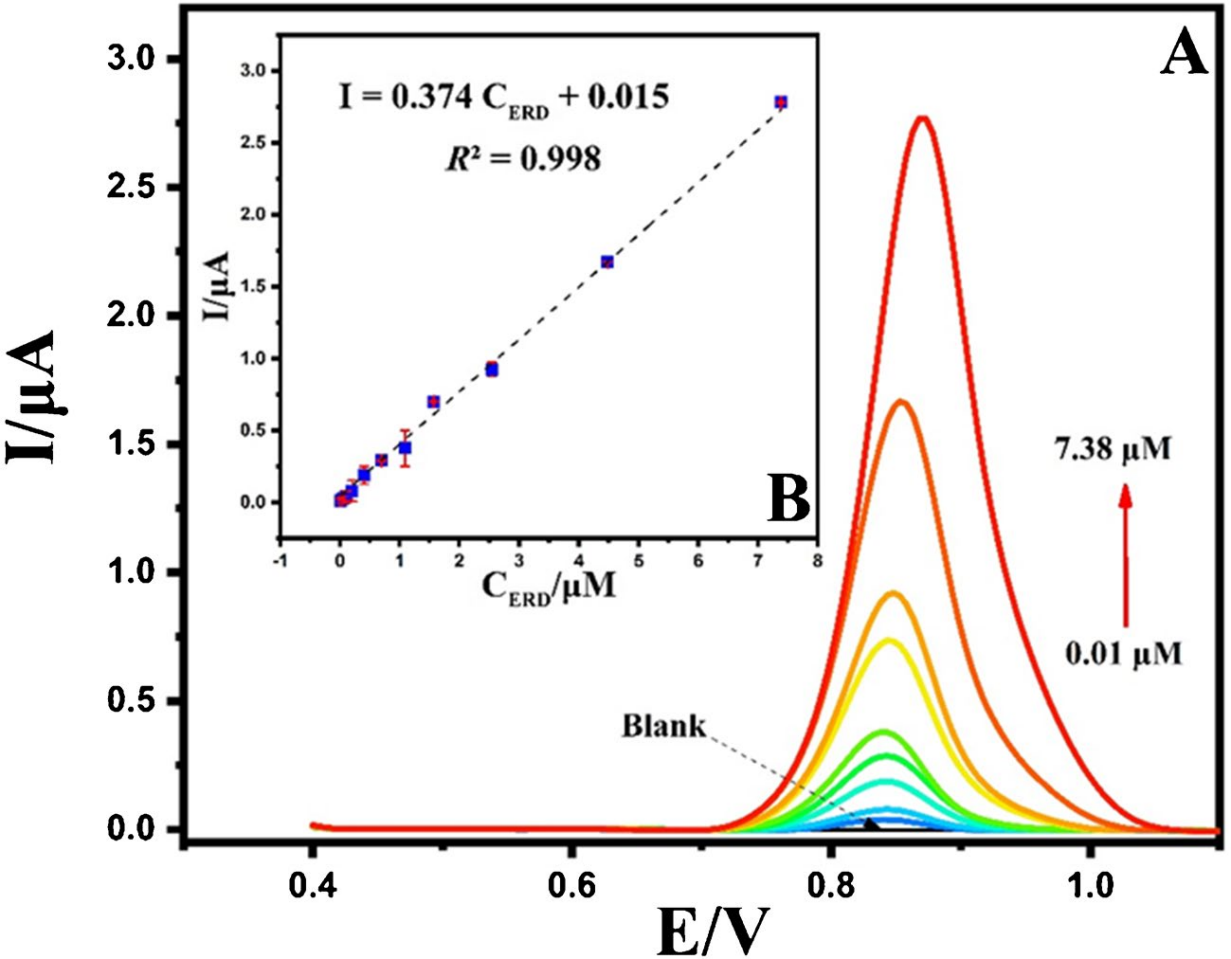
DPVs of different concentrations of ERD from 0.01 to 7.38 μM (**A**), the graph of Ipa versus C_ERD_ (**B**)

In order to comprehensively evaluate the analytical prowess of the developed sensor, a comprehensive investigation was undertaken, involving a systematic analysis across a range of ERD concentrations. This assessment was conducted using the precise DPV method under optimized conditions (Fig. 16A). Subsequently, a meticulously crafted calibration curve was generated by correlating Ipa with escalating concentrations of ERD (Fig. 16B).

The findings elucidated a robust linear relationship within the concentration range of 0.01–7.38 μM, substantiating the sensor’s aptitude for discerning ERD concentrations with a high degree of precision. The linear regression formula characterizing this association was derived as I = 0.374 C_ERD_ + 0.015 (R^2^ = 0.998). LOD (limits of detection) and LOQ (limits of quantification) were judiciously established by employing the equation LOD = 3 s.m^−1^ and LOQ = 10 s.m^−1^, severally. These formulations facilitate a nuanced evaluation of the analyte’s minimum discernible and measurable concentrations. Within the scope of our investigation, LOD and LOQ results were ascertained to be 3.36 nM and 11.2 nM (Table 3), severally. The discernments mentioned furnish crucial perspectives into the heightened sensitivity and accuracy intrinsic to the designed sensor, underscoring its potential for detecting ERD at exceedingly low concentrations, a pivotal attribute in advancing its utility for pharmaceutical analysis and clinical applications.

**Table 3:** Analytical performance data for the proposed method

Parameter	Value
Measured potential (V)	0.843
Linearity range (μg/mL)	0.01–7.38
Regression equation	I = 0.374 C_ERD_ + 0.015
Determination coefficient (R^2^)	0.998
Intercept	0.015
Slope	0.374
SE of intercept	0.015
SE of slope	0.005
LOD (ng/mL)	3.36
LOQ (ng/mL)	11.2
*SE, standard error	

Table 4 provides a comparison of Erdafitinib concentration determination, considering the linear range and LOQ, with prior findings documented in the literature [15–20]. The current methodology is straightforward and eliminates the need for pre-treatment procedures or laborious and chemical-intensive reactions such as derivatization. Therefore, it is noteworthy that this method significantly reduces preparation costs and provides rapid detection compared to existing standard procedures. Additionally, as can be seen from the table, the method is superior to most studies in the literature in terms of linear range and LOQ. This suggests that the current voltammetric method is more sensitive and offers a sufficiently wide linear range for ERD determination

**Table 4:** Comparison between the proposed method for determination of ERD to previously reported methods

Method	Linear range (ng/mL)	LOD (ng/mL)	LOQ (ng/mL)	Analytical performance					Application	Ref
				Slope	Intercept		R^2^			
HPLC–UV	50–2000	-	50	0.9597		− 0.0013	≥ 0.992	Mouse plasma		[15]
HPLC–UV	500–50.000	200.0	500.0	10.076		0.5218	0.999	Laboratory-prepared Tablets		[16]
LC–MS/MS	3.0–1000	-	3.0	0.0051		0.0078	≥ 0.991	Human plasma		[17]
UPLC-MS/MS	1.0–500	-	1.0	0.9009		0.3741	0.9993	Beagle dogs plasma		[18]
UPLC-MS/MS	0.5–1000	-	0.5	-		-	> 0.997	Human plasma		[19]
Spectrofluorimetry	50–800	14.36	43.50	0.4794		− 3.6071	0.9998	Laboratory-prepared Tablets, Human plasma		[20]
DPV on GCE	10–7380	3.36	11.2	0.374		0.015	0.998	Synthetic urine, Tablets (Balversa®)		Current study

### The evaluation of some validation parameters for the modified electrode.

In evaluating the repeatability of the newly devised NPCS/GCE, an exhaustive examination involving 9 successive cycles was conducted, and the %RSD (relative standard deviation) was meticulously ascertained, as illustrated in Figure S6A. The resulting %RSD for all ninr cycles was remarkably low at 2.66%, providing clear evidence of the exceptional repeatability of the NPCS/GCE.

Furthermore, a meticulous assessment of the reproducibility of the NPCS/GCE was conducted through the fabrication of 6 electrodes under identical conditions. DPV signals were systematically recorded for each of these electrodes, as delineated in Figure S6B. The observed current variations exhibited an impressively low RSD% of 2.13%, highlighting the outstanding reproducibility demonstrated by the NPCS/GCE.

Additionally, with its noteworthy repeatability and reproducibility, the discerningly developed sensor is imperative to showcase a pronounced selectivity specifically tailored toward the designated target analyte. This criterion, when met, further enhances the efficacy and applicability of the developed NPCS/GCE in demanding analytical scenarios, thereby solidifying its standing as a sophisticated and reliable sensing platform within the realm of pharmaceutical sciences.

In this study, to verify the selectivity and application of the voltammetric approach, interference studies were performed using chemicals frequently included in drugs and biological fluids. These chemicals were vitamin C (ascorbic acid(AA)), C_5_H_4_N_4_O_3_ (uric acid(UA)), D-Glc(D-G), L-Arg(L-A)[109], L-Met(L-M)[110], KCl (potassium chloride), Na_2_SO_4_ (sodium sulfate) and KNO_3_ (potassium nitrate) (Figure S7). The experiments were carried out under optimum conditions where ERD was kept constant at 1 μM, and the interfering substances were used 100-fold. The results, revealed through thorough experimentation, distinctly show no significant impact on the electrochemical currents related to ERD. The observed stability is represented by an RSD% confined to a mere 2.55%.Thus, the developed sensor exhibits high repeatability, reproducibility, and sensitivity, further enhancing its efficacy in analytical endeavors.

### Real sample analysis

The efficacy of the NPCS/GCE was systematically evaluated in the context of ERD assay, employing commercially available synthetic human urine and tablet forms as representative specimens. Employing the standard addition technique, DPV was judiciously implemented to quantitatively discern the concentration of ERD within the authentic samples. Upon examining the results presented (Table 5), it is clear that the created electrode shows a notable capability for directly detecting ERD in real samples. The recorded recoveries, spanning a range from 98.40 to 101.04% for urine and 100.34 to 102.24% for tablet formulations, underscore the robustness and reliability of the NPCS/GCE in discerning ERD concentrations within these complex matrices. Consequently, it can be unequivocally affirmed that the suggested voltammetric method exhibits a high degree of accuracy, thereby substantiating its proficiency in the precise determination of ERD concentrations in real-world samples. This outcome holds particular significance in the realm of pharmaceutical sciences, signifying the pragmatic applicability of the NPCS/GCE as a potent tool in pharmaceutical analysis.

**Table 5:** Analysis of synthetic human urine samples and dosage forms (as tablets) in the presence of ERD

Sample	Added (µM)	Found (µM) ^a^	RSD (%)	Recovery (%)
Urine	0.2	0.202	0.62	101.04
	0.3 0.4	0.295 0.404	0.57 0.64	98.40 100.98
Tablet	0.2 0.3 0.4	0.204 0.302 0.401	2.25 0.89 1.30	102.24 100.85 100.34

^a^Mean value derived from three replicated measurements

## Conclusion

In this study, we have introduced a novel electrochemical method for quantifying Erdafitinib, an anti-cancer pharmaceutical agent. This innovative approach utilizes an electrochemical sensor modified with a ZIF-12-based NPCS produced via the molten salt-assisted method. To begin with, the interpenetrated porous structure facilitates the transportation of substances. Furthermore, the electronic conductivity and wettability of NPC material are enhanced through nitrogen doping. Furthermore, the method utilizing molten salt as an aid enhances the extent of graphitization in NPC materials by providing guidance for the carbon organization. These benefits not only encourage the advancement of porous carbon as a material for electrode modification in medical applications, but they may also inspire the development of alternative electrode materials.

The material’s unique microstructure contributes to its exceptional electrochemical characteristics. The incorporation of the NPCS nanocomposite significantly augmented the surface area of the GCE and enhanced the electrical conductivity of the fabricated sensors. Under optimal conditions, NPCS/GCE demonstrated heightened sensitivity in the determination of the anticancer drug ERD across a linear range of 10 nM to 7.38 μM, with an impressively low detection limit of 3.36 nM. Moreover, NPCS/GCE exhibited successful application in the determination of ERD in real samples, showcasing acceptable recovery data ranging from 98.40 to 101.04% for tablets and 100.34 to 102.24% for urine samples. The resulting sensor, compared to other methods, demonstrates outstanding performance characteristics, including heightened sensitivity and precise selectivity, simplicity, and cost-effectiveness, positioning it as a promising tool for ERD analysis in biological samples and pharmaceutical formulations. Moreover, this marks the first occurrence of electrochemical analysis conducted on ERD. Subsequent studies may explore extending the use of this electrochemical technique with additional anti-neoplastics.

## Supplementary information

For a more comprehensive understanding and detailed insights, it is recommended that readers refer to the Electronic Supplementary Information.

### Supplementary Information

Below is the link to the electronic supplementary material.

Supplementary file1 (DOCX 982 KB)

## Data Availability

Data will be made available on request.
