# Supplementary material for: Pioneering electrochemical detection unveils erdafitinib: a breakthrough in anticancer agent determination
Source: Mikrochim Acta. 2024 Mar 27;191(4):221. doi: 10.1007/s00604-024-06318-z (PMC10973028; doi:10.1007/s00604-024-06318-z)
Supplement: Supplementary file 1 — Supplementary file1 (DOCX 982 KB) [file 604_2024_6318_MOESM1_ESM.docx]

**Supplementary Information**

**Pioneering Electrochemical Detection Unveils Erdafitinib: A Breakthrough in Anticancer Agent Determination**

Merve Hatun Yildir^a,b;*^, Asena Ayse Genc^a,b^, Nevin Erk^a;*^, Wiem Bouali^a,b^, Nesrin Bugday^c^, Sedat Yasar^c^, Ozgur Duygulu^d^

^a^Ankara University, Faculty of Pharmacy, Department of Analytical Chemistry, 06560, Ankara, Turkey

^b^Ankara University, Graduate School of Health Sciences, 06110, Ankara, Turkey

^c^ İnonu University, Department of Chemistry, 44280, Malatya, Türkiye

^d^TÜBİTAK Marmara Research Center, Materials Technologies, TÜBİTAK Gebze Campus, 41470 Gebze, Kocaeli, Turkey

*Corresponding authors: [erk@pharmacy.ankara.edu.tr](mailto:erk@pharmacy.ankara.edu.tr) and [ecz.merveyildir@gmail.com](mailto:ecz.merveyildir@gmail.com)

**Reagents**

Erdafitinib (99.94 %) was obtained from Selleckchem (TX, USA). Potassium hexacyanoferrate (III) (K_3_Fe(CN)_6_, (99.5 %), L-ascorbic acid (99.0 %), glucose (99.5 %), dopamine hydrochloride (99.0 %), uric acid (99.0 %), L-arginine (98.0%), L-cysteine (97.0%), potassium chloride (KCl), potassium nitrate (KNO_3_), Sodium sulfate (Na_2_SO_4_), acetaminophen, Sodium chloride (NaCl) and synthetic human urine were supplied from Sigma Aldrich Co. (Germany). Ethanol (C_2_H_5_OH), Acetonitrile (CH_3_CN) (99.9 %), sodium hydroxide (NaOH) were supplied from Merck (Darmstadt, Germany). Benzimidazole (BIM), Cobalt acetate tetrahydrate ((CH_3_COO)_2_Co.4H_2_O), and ammonium hydroxide (NH_3_, 28–30% aqueous solution) were purchased from Alfa Aesar. All chemicals are analytical reagents and are used as received without any further purification.

0.1 M phosphate-buffered saline (PBS) used in this study was prepared using analytical grade NaH_2_PO_4_.H_2_O (≥98%) and KH_2_PO_4_ (≥98%). Britton-Robinson buffer was prepared using boric acid, phosphoric acid, potassium chloride, and acetic acid solutions. The pH values ​​of these buffers were adjusted with the help of 2.0 M NaOH and 2.0 M HCl. Ferricyanide solution was prepared using 5 mM K_3_[Fe(CN)_6_] (99.5%) and 0.1 M KCl.

All solution preparation processes were carried out using Millipore water with a resistivity of 18.2 MΩ. All experiments were conducted at the same temperature (~ 24 °C).

**Apparatus**

XRD analysis of the prepared materials was carried outusing Rigaku Rint 2000 X-ray Diffractometer between 2 and 80 /min with a scan rate of 2/min. The morphology of the materials were examined by a scanning electron microscope (SEM). SEM investigations were performed at 15 kV by JEOL JSM 6510LV SEM with Oxford Instruments Model 7260 EDS system. Specimens were investigated by JEOL JEM 2100 High Resolution Transmission Electron Microscope (LaB6 filament) operated at 200 kVand equipped with an Oxford Instruments X-Max 80T Energy Dispersive Spectrometer (EDS) system. Carbon support film coated copper TEM grids (Electron Microscopy Sciences, CF200-Cu, 200 mesh) were used. Images were taken by Gatan Model 833 Orius SC200D CCD Camera and also by Gatan Model 794 Slow Scan CCD Camera. For digital images, Gatan Microscopy Suite (GMS) 2 software was used. For diffraction pattern analysis and crystallography purposes CrystBox software was used [1]. The elemental composition and phase structure were analyzed by X-ray photoelectron spectroscopy (XPS) and were recorded using a Specs-Flex XPS instrument in the range of 200-4000 eV.

Electrochemical measurements were performed using the Metrohm-Autolab potentiostat/galvanostat system (PGSTAT128 N, The Netherlands). In this research, Ag/AgCl (in saturated KCl), platinum wire, and glassy carbon electrode (GCE) were used as reference, auxiliary, and working electrodes, respectively. pH measurements for the prepared phosphate buffer solutions were performed using a HANNA pH meter (Edge® Multiparameter pH Meter - HI2020).


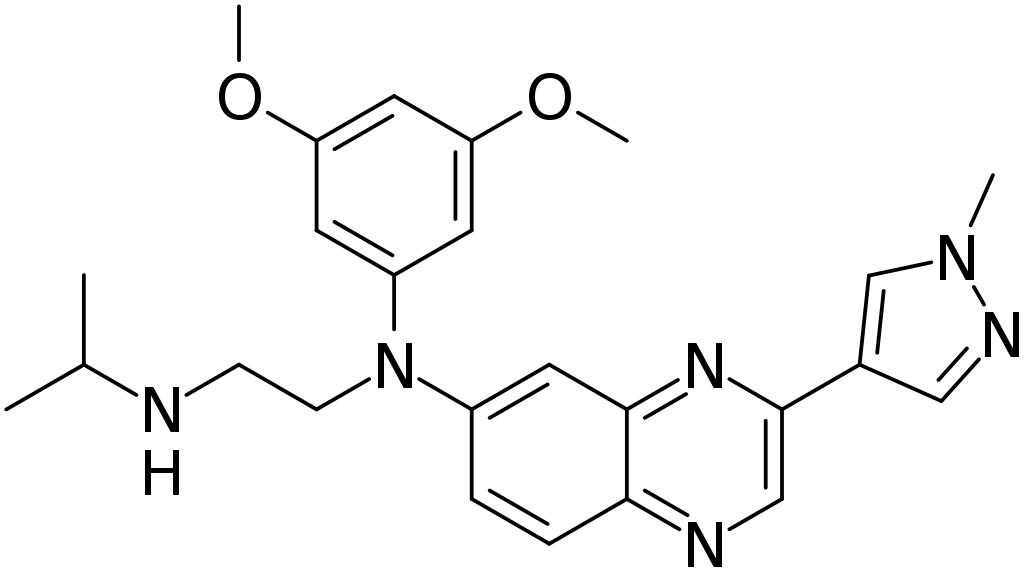


**Figure S1.** Molecular structure of Erdafitirib.

**Figure S2.** The graphic of Williamson-Hall method for the calculate average crystallite size

**
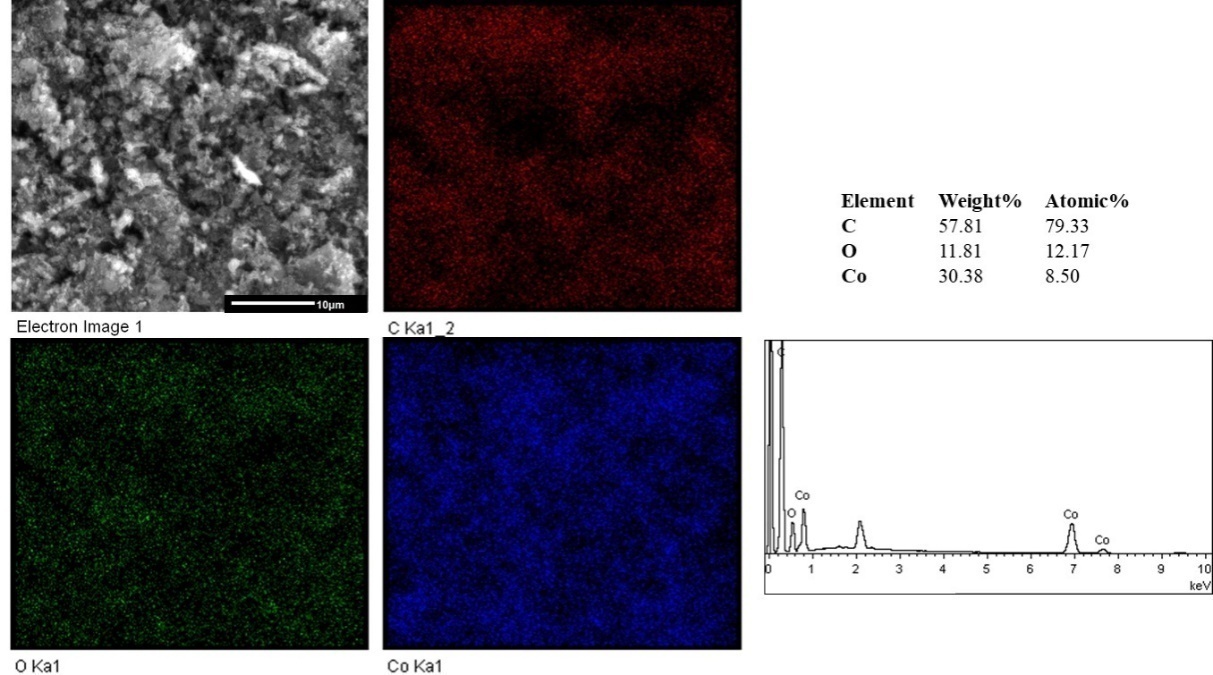
**

**Figure S3.** SEM image,EDS mapping, EDS results and spectrum of NPCS sample.

**Table S1.** The content of elements according to XPS analyse in NPCS

| **XPS** | **At%** |
| --- | --- |
| O 1s | 17.54 |
| C 1s | 75.86 |
| N 1s | 1.20 |
| Co 2p | 5.41 |

$Ipa=2.69 \times{10}^{5}n^{\frac{3}{2}}{AD}^{\frac{1}{2}}Cv^{\frac{1}{2}}$ (Equation S1)

(It is expressed as $Ipa$: anodic peak current, $n$: number of electrons, $A$: electroactive surface area (cm^2^), $D$: diffusion coefficient (cm^2^/s^1^), $C$: concentration (mol/cm^3^), $v$: scan rate (mV/s^1^).)

$Epa=E^{0}+\left( \frac{RT}{anF} \right)ln\left( \frac{RTK^{0}}{anF} \right)+\left( \frac{RT}{anF} \right)lnv$ (Equation S2)


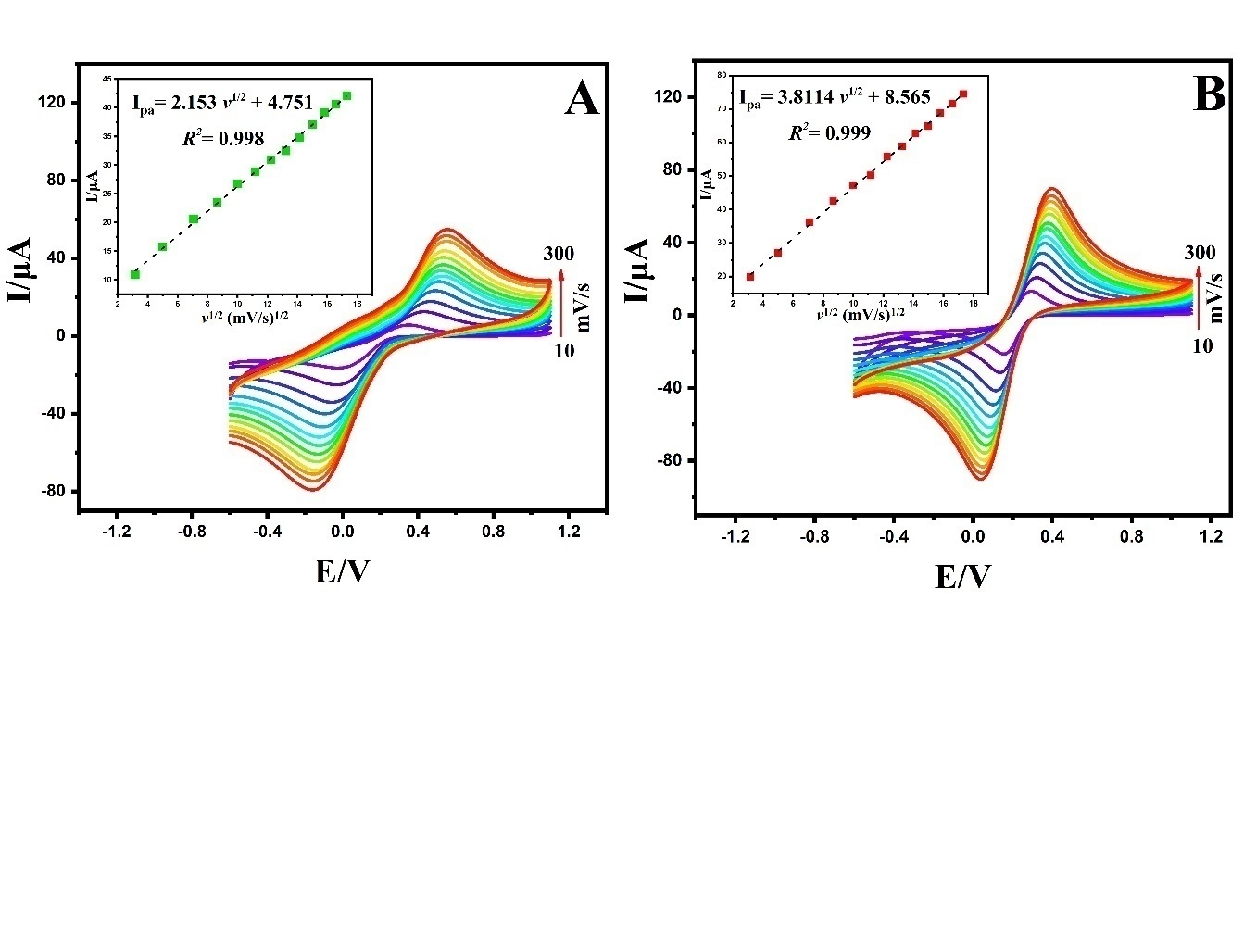


**Figure S4.**The recorded CV curves at various scan rates(10 to 300 mV/s) in the presence of 5.0 mM [Fe (CN) _6_]^3−/4−^containing 0.1 M KCl on (A); and bare GCE, (B); NPCS/GCE.


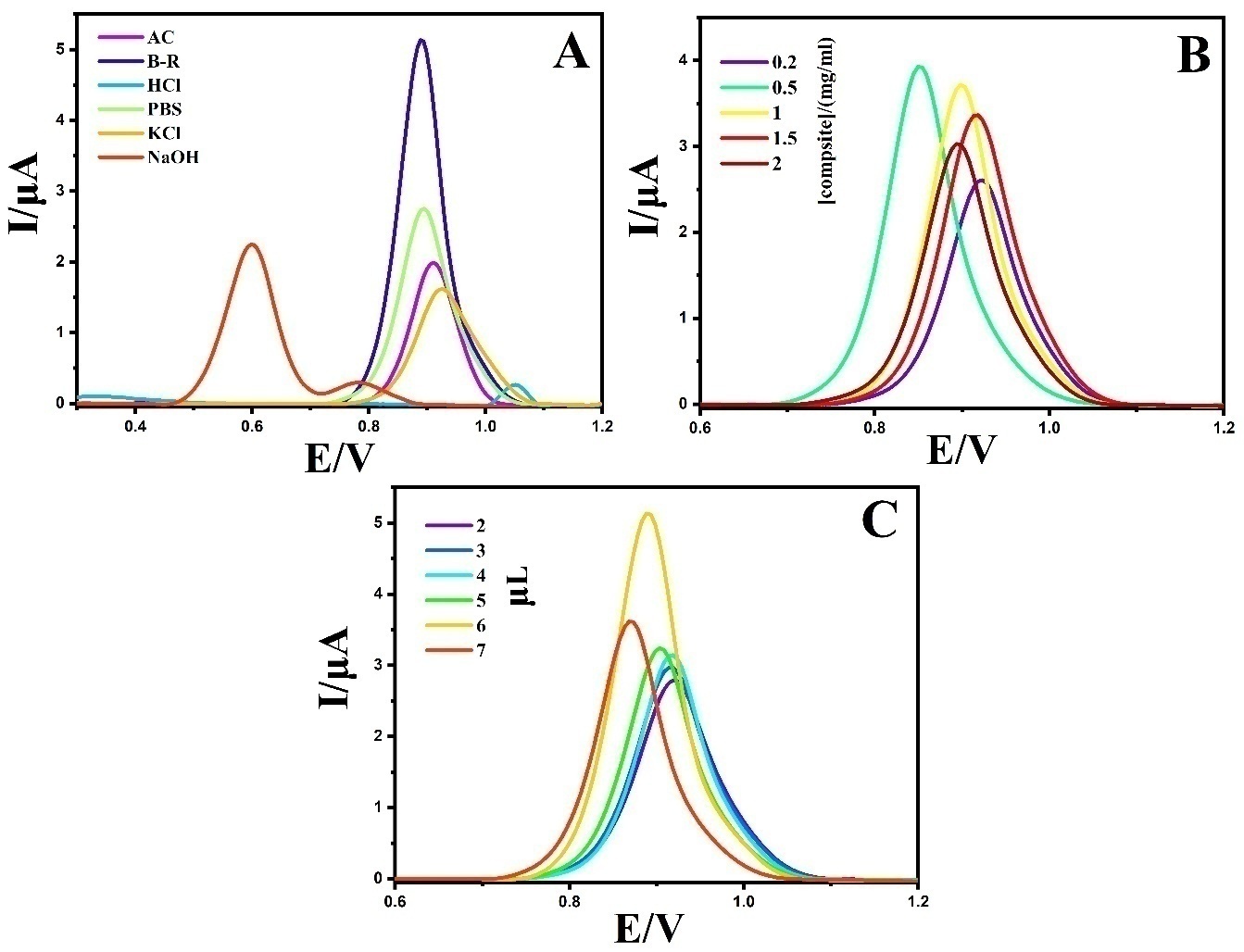


**Figure S5.** Influence of (A) supporting electrolyte; (B) concentration; and (C) volume of NPCS composite on the peak response currents of 10 μM ERD.

**
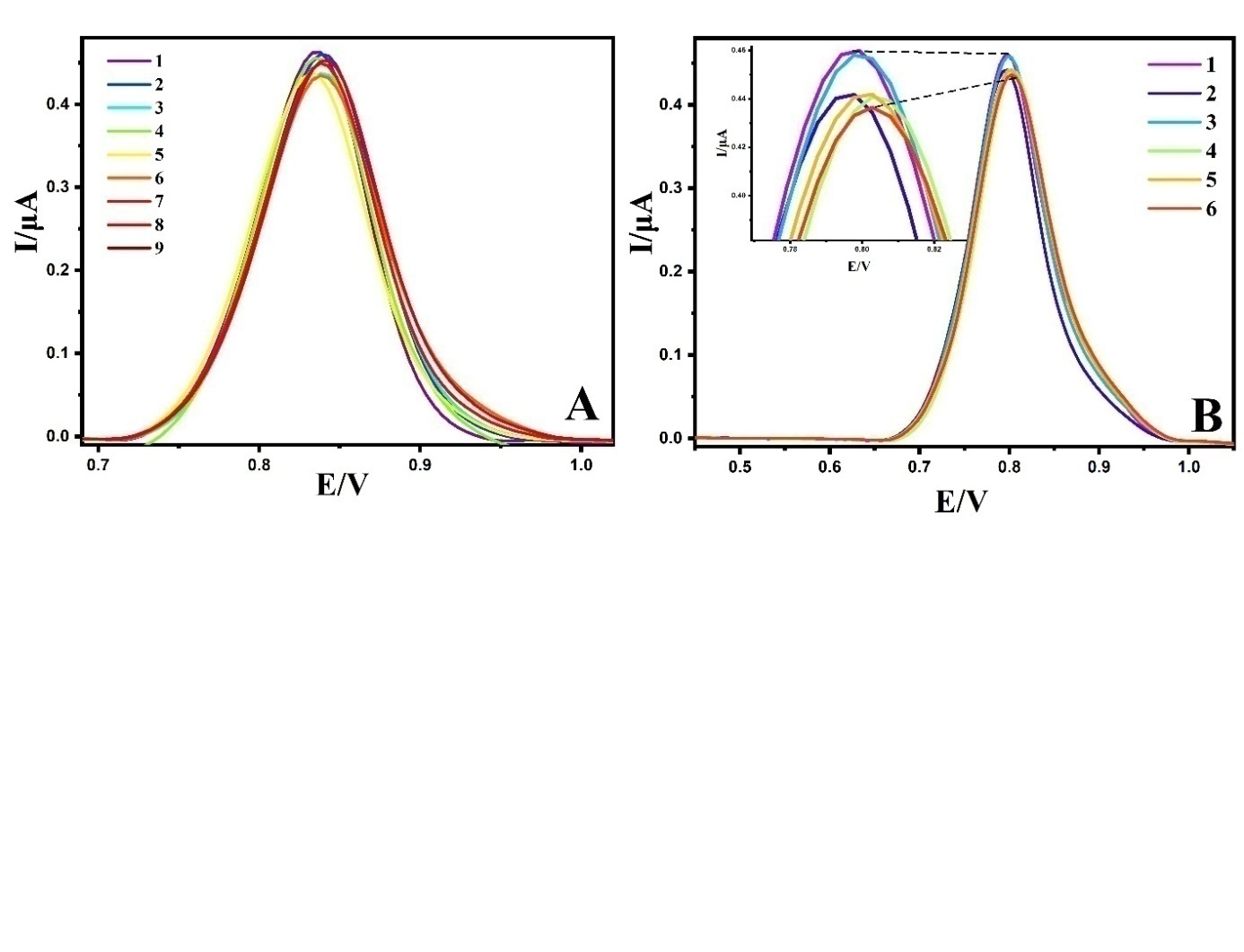
**

**Figure S6.** Repeatability(A), and reproducibility (B) of 1.0 μM ERD at NPCS/GCE in B-R buffer (pH 6.0).


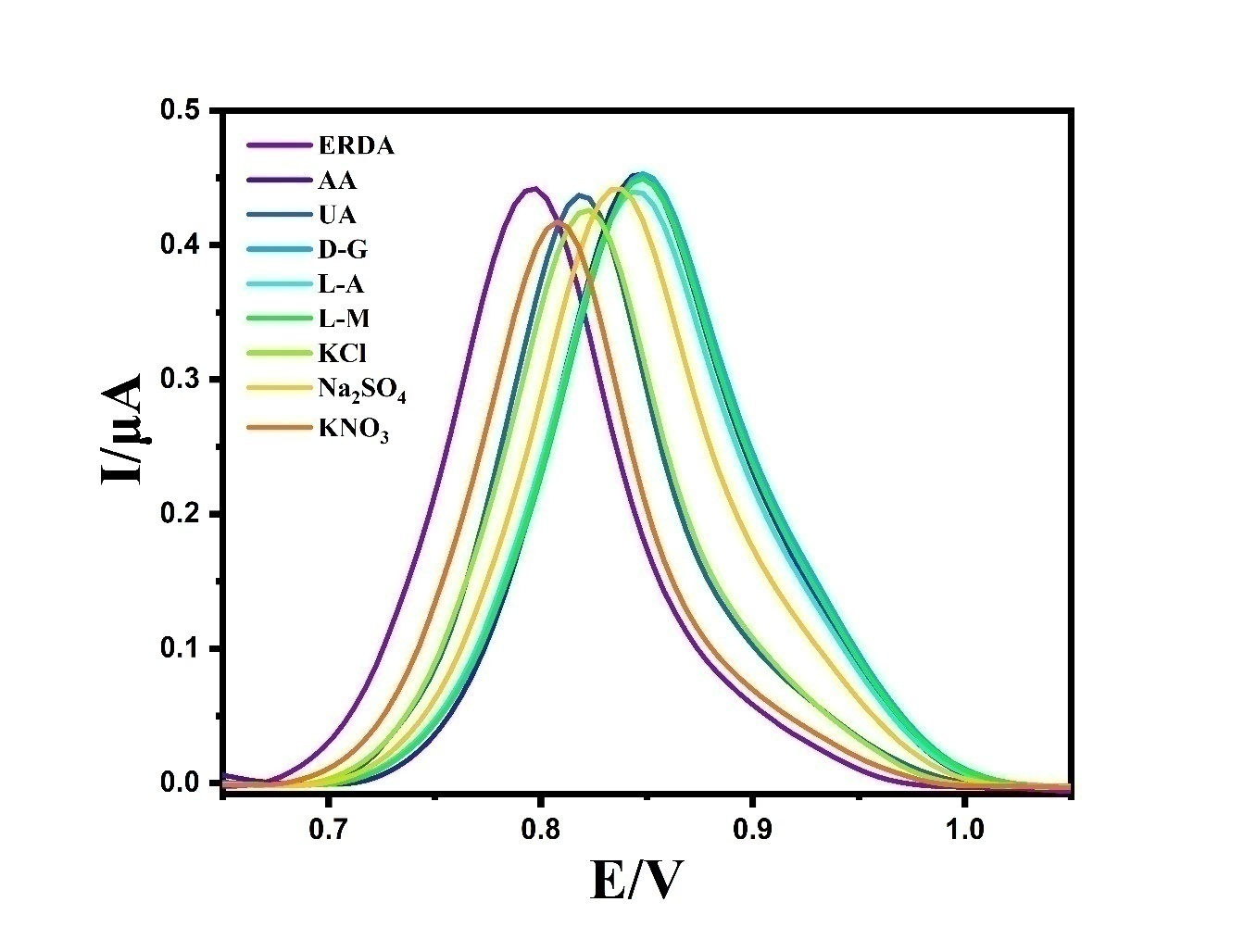


**Figure S7.** Illustration of Selectivity of 1.0 μM ERD at NPCS/GCE in pH 6.0 B-R buffer.

**Reference**

1. Klinger, M., *CrysTBox-crystallographic toolbox.* Institute of Physics of the Czech Academy of Sciences. Czech Republic, Prague, 2015. ISBN 978-80-905962-3-8. http://www.fzu.cz/~klinger/ crystbox.pdf
